# Economic Evaluation of an Internet-Based Preventive Cognitive Therapy With Minimal Therapist Support for Recurrent Depression: Randomized Controlled Trial

**DOI:** 10.2196/10437

**Published:** 2018-11-26

**Authors:** Nicola S Klein, Claudi LH Bockting, Ben Wijnen, Gemma D Kok, Evelien van Valen, Heleen Riper, Pim Cuijpers, Jack Dekker, Colin van der Heiden, Huibert Burger, Filip Smit

**Affiliations:** 1 Department of Psychology University of Groningen Groningen Netherlands; 2 Top Referent Traumacentrum GGZ Drenthe Beilen Netherlands; 3 Department of Psychiatry Amsterdam University Medical Centers University of Amsterdam Amsterdam Netherlands; 4 Department of Public Mental Health Trimbos Institute Utrecht Netherlands; 5 Care and Public Health Research Institute School for Public Health and Primary Care Department of Health Services Research Maastricht University Maastricht Netherlands; 6 Ambulante Forensische Psychiatrie Noord GGZ Drenthe Emmen Netherlands; 7 Department of Geriatrics University Medical Center Utrecht Utrecht Netherlands; 8 Department of Clinical, Neuro and Developmental Psychology Vrije Universiteit Amsterdam Netherlands; 9 Amsterdam Public Health VU University Medical Center Amsterdam Netherlands; 10 GGZ inGeest Amsterdam Netherlands; 11 Research Department Arkin Mental Health Institute Amsterdam Netherlands; 12 Department of Psychology Erasmus University Rotterdam Rotterdam Netherlands; 13 PsyQ Mental Health Care Center Rotterdam Netherlands; 14 Department of General Practice University Medical Center Groningen Groningen Netherlands

**Keywords:** major depressive disorders, recurrence, cognitive therapy, internet, prevention, cost effectiveness

## Abstract

**Background:**

Major depressive disorder (MDD) is highly recurrent and has a significant disease burden. Although the effectiveness of internet-based interventions has been established for the treatment of acute MDD, little is known about their cost effectiveness, especially in recurrent MDD.

**Objectives:**

Our aim was to evaluate the cost effectiveness and cost utility of an internet-based relapse prevention program (mobile cognitive therapy, M-CT).

**Methods:**

The economic evaluation was performed alongside a single-blind parallel group randomized controlled trial. Participants were recruited via media, general practitioners, and mental health care institutions. In total, 288 remitted individuals with a history of recurrent depression were eligible, of whom 264 were randomly allocated to M-CT with minimal therapist support added to treatment as usual (TAU) or TAU alone. M-CT comprised 8 online lessons, and participants were advised to complete 1 lesson per week. The economic evaluation was performed from a societal perspective with a 24-month time horizon. The health outcomes were number of depression-free days according to *Diagnostic and Statistical Manual of Mental Disorders, Fourth Edition*, (DSM-IV) criteria assessed with the Structured Clinical Interview for DSM-IV axis I disorders by blinded interviewers after 3, 12, and 24 months. Quality-adjusted life years (QALYs) were self-assessed with the three level version of the EuroQol Five Dimensional Questionnaire (EQ-5D-3L). Costs were assessed with the Trimbos and Institute for Medical Technology Assessment Questionnaire on Costs Associated with Psychiatric Illness (TiC-P). Incremental cost-effectiveness ratios were calculated and cost-effectiveness planes and cost-effectiveness acceptability curves were displayed to assess the probability that M-CT is cost effective compared to TAU.

**Results:**

Mean total costs over 24 months were €8298 (US $9415) for M-CT and €7296 (US $8278) for TAU. No statistically significant differences were found between M-CT and TAU regarding depression-free days and QALYs (*P*=.37 and *P*=.92, respectively). The incremental costs were €179 (US $203) per depression-free day and €230,816 (US $261,875) per QALY. The cost-effectiveness acceptability curves suggested that for depression-free days, high investments have to be made to reach an acceptable probability that M-CT is cost effective compared to TAU. Regarding QALYs, considerable investments have to be made but the probability that M-CT is cost effective compared to TAU does not rise above 40%.

**Conclusions:**

The results suggest that adding M-CT to TAU is not effective and cost effective compared to TAU alone. Adherence rates were similar to other studies and therefore do not explain this finding. The participants scarcely booked additional therapist support, resulting in 17.3 minutes of mean total therapist support. More studies are needed to examine the cost effectiveness of internet-based interventions with respect to long-term outcomes and the role and optimal dosage of therapist support. Overall, more research is needed on scalable and cost-effective interventions that can reduce the burden of recurrent MDD.

**Trial registration:**

Netherlands Trial Register NTR2503; http://www.trialregister.nl/trialreg/admin/rctview.asp?TC=2503 (Archived by WebCite at http://www.webcitation.org/73aBn41r3)

## Introduction

In 2016, an estimated 268 million individuals worldwide were affected with a depressive disorder [1]. Major depressive disorder (MDD) is a highly recurrent disorder [2] with a substantial disease burden [1,3] and formidable economic costs due to health care use and productivity losses [4-7].

To alleviate the burden of MDD, psychological interventions and/or antidepressants are recommended for the acute phase of MDD and as continuation/maintenance therapy to prevent relapse and recurrence [8]. However, health care resources are limited and many individuals fail to seek treatment [9-12]. Because of their flexible and accessible nature, internet-based interventions could be a viable cost-effective solution that reaches a large number of at-risk individuals. The effectiveness of internet-based interventions in acute and residual MDD has been established [13-17], with small to moderate effect sizes for interventions without therapist support and higher effect sizes with therapist support [13,15]. To date, only one study examined the long-term effects of an internet-based relapse prevention program for depression [18] and no study has examined its cost effectiveness. Only a single study aimed at the prevention of MDD examined the cost effectiveness of an internet-based intervention but this study aimed to prevent the first onset of MDD [19]. Thus, little is known about the cost effectiveness of internet-based relapse prevention for recurrent MDD. More information is needed on the health impact and economic costs to inform policy makers and health care providers.

In our randomized controlled trial (RCT), we examined the clinical effectiveness of an internet-based relapse prevention program (mobile cognitive therapy, M-CT) added to treatment as usual (TAU) compared to TAU alone in remitted individuals with recurrent MDD. Results showed that M-CT added to TAU was slightly but not significantly superior to TAU alone after 24 months in terms of cumulative relapse/recurrence rate, number of depressive relapses, and depressive symptoms [20]. In this study, we evaluated the cost effectiveness and cost utility of M-CT to see if the economic case could be made for this low-cost and highly scalable intervention that was added to TAU. We hypothesize that M-CT added to TAU is cost effective compared with TAU alone as it might generate slightly better health outcomes and thereby lower costs for other mental health services.

## Methods

### Study Design

The economic evaluation was performed alongside a single-blind parallel group 2-arm RCT in which 288 participants aged between 18 and 65 years were eligible, of whom 264 were randomized to either M-CT added to TAU or TAU alone. This trial was registered at Nederlands Trial Register [NTR2503] and approved by Stichting Medisch-Ethische Toetsingscommissie Instellingen Geestelijke Gezondheidszorg, an independent medical ethics committee. The results were reported according to the Consolidated Standards of Reporting Trials–EHEALTH checklist [21] (Multimedia Appendix 1). The economic evaluation was conducted and reported according to the Consolidated Health Economic Evaluation Reporting Standards statement (Multimedia Appendix 2). The study design and results are described in detail elsewhere [20,22] but are summarized briefly here.

### Participants and Procedure

The participants were recruited via media, general practitioners, and mental health care institutions and were included between mid-September 2010 and August 2013 after providing a written informed consent. To be included, the following criteria had to be met: (1) a history of at least 2 major depressive episodes (MDEs) according to *Diagnostic and Statistical Manual of Mental Disorders, Fourth Edition,* (DSM-IV) criteria assessed with the Structured Clinical Interview for DSM-IV Disorders (SCID-I) [23] of which the latest MDE occurred within the last 2 years and (2) currently remitted for at least 2 months according to the SCID-I and a score of ≤10 on the Hamilton Rating Scale for Depression (HRSD) [24]. Exclusion criteria were current or past (hypo)mania, bipolar or psychotic disorder, alcohol or drug abuse, or a predominant anxiety disorder. Independent psychologists or research assistants interviewed the potential participants for inclusion and exclusion criteria. Our initial criterion of having experienced at least 2 depressive episodes within 5 years was discarded, as individuals with multiple episodes over a longer period of time are also at risk [25]. We examined whether this affected our primary outcomes, but this was not the case.

### Randomization and Masking

Randomization was planned to be stratified by type of aftercare and number of MDEs, but eventually simple randomization was carried out due to a programming error. The participants were randomized (allocation ratio 1:1) by an independent researcher not otherwise involved in the study who was masked for clinical characteristics and who used computer-generated numbers generated in Stata software (StataCorp LLC). An independent researcher not involved in the follow-up interviews assigned the participants to the treatment conditions. The participants were not blinded to treatment allocation due to the nature of the intervention. The interviewers were unaware of the participants’ treatment allocation, and the participants were instructed not to inform the interviewer of their treatment allocation. The assessor was replaced by another independent assessor in case the randomization was broken.

### Interventions

M-CT is based on preventive cognitive therapy (PCT) [26], a face-to-face therapy that protects against relapse/recurrence in remitted individuals [27-29]. Bockting and Van Valen developed the content of M-CT [30], and it was built into the ePlatform of the Trimbos Institute, a nonprofit organization in the Netherlands with a focus on issues related to mental health and addiction. Participants from previous relapse prevention studies and a patients’ association for depression (Depressievereniging) were involved in the development of the research question, outcome measures, design, development, and implementation of M-CT. The content of the program remained unaltered during the evaluation period and logfile analysis was used to monitor the use of the intervention. The program was free of charge for the participants, and an independent researcher provided participants with usernames and passwords to log in. M-CT comprised 8 online modules with minimal therapist support and continued mobile mood monitoring using text messages. The participants were advised to work on one lesson each week and were offered a minimum of 2 and a maximum of 4 telephone contacts with a therapist (maximum duration: 30 minutes per contact). Two therapist contacts were prebooked and 2 optional contacts could be booked by the participants additionally. Participating therapists were supervised by an experienced clinical psychologist. The primary task of the therapists was to work through the M-CT program. The participants received a reminder by email or text message if they did not log in to the website for 6 weeks. Feedback from the participants on the intervention was obtained by giving them the opportunity to evaluate each specific lesson. Participants randomized to M-CT and TAU continued to receive usual care, which could include, for example, antidepressants, counseling, or no care.

### Costs

The economic evaluation was performed according to the Dutch guidelines [31] in which a societal perspective is recommended, implying that costs both inside and outside the health care sector are assessed. Health care costs included medication use and inpatient, outpatient, and primary care. Costs directly related to the M-CT intervention included costs of training and supervision of therapists during the study, the duration of contacts between participants and therapists (telephone and email contact), and costs related to information and communication technologies. The last category mainly consisted of costs related to developing, upgrading, and maintaining the M-CT website. In addition, various types of costs outside the health care sector were examined. Patient and family costs included informal care (ie, the monetary valuation of time invested by relatives or acquaintances in helping or assisting the participant), travel expenses, and psychiatric home care. Costs of productivity losses due to illness-related absence from work were estimated as were costs related to changes in efficiency while at work and changes in the amount of voluntary (unpaid) work conducted by the participants. Costs of productivity losses were estimated with the friction cost method [32]. This method takes the employer’s perspective and calculates the time (the friction period) until another employee has replaced the worker that is absent.

Cost data were collected with the Trimbos and Institute for Medical Technology Assessment Questionnaire on Costs Associated with Psychiatric Illness (TiC-P) [33]. This questionnaire was administered online to all participants every 3 months, starting at baseline. Since there was variation in the maximum number of days medication use could be filled out by the participants, we extrapolated all medication use to 3 months. In order to facilitate comparisons with other economic evaluations, unit prices (ie, the price of one unit of each included cost type) were based on Dutch standard prices for the year 2014 [34]. Full economic cost prices of used resources were computed when standard prices were not available.

### Outcomes

The economic evaluation comprised cost-effectiveness and cost-utility analyses. The health outcome measure of the cost-effectiveness analysis was the number of depression-free days based on DSM-IV criteria assessed with a telephone version of the SCID-I after 3, 12, and 24 months.

Quality-adjusted life years (QALYs) were used as a health outcome measure of the cost-utility analysis using the area under the curve method. The QALY is a health measure that combines quality of life and the amount of time spent in a health condition, where one QALY is equal to 1 year lived in perfect health. The quality component of the QALY was derived from the three level version of the EuroQol Five Dimensional Questionnaire (EQ-5D-3L) administered online at 3-month intervals starting from baseline [35] by using Dutch tariffs to obtain utilities for specific health states [36]. The EQ-5D-3L is a commonly applied self-administered instrument that measures the generic health status and consists of 5 questions covering the following 5 dimensions: mobility, self-care, usual activities, pain/discomfort, and anxiety/depression. Both costs and health outcomes were discounted at 1.5% for health outcomes and 4% for costs according to the Dutch guidelines [31].

### Economic Evaluation

The power calculation of the primary outcome is described elsewhere [20,22]. Since the study was only powered to detect differences in health outcomes and not in costs, as in most economic evaluations, we used probabilistic and medical decision-making techniques to draw inferences about the cost effectiveness. The intention-to-treat principle was used, in which all participants were analyzed according to their randomized condition, irrespective of their actual treatment. In our main analysis, we used multiple imputations by chained equations with predictive mean matching to account for missing data. The use of this technique may avoid bias associated with complete case analyses and makes optimal use of available data. Baseline variables predictive of clinical and cost outcomes and of a variable being missing were incorporated in the imputation model as recommended by White et al [37] to enhance the precision of the model and correct for possible bias. To account for participants with extremely high costs resulting in unstable imputation estimates, winsorizing was used for the main analyses. Using winsorizing, extreme values are instead replaced by certain percentiles, in this case the 97.5th percentile as opposed to trimming in which the extreme values are merely deleted [38,39].

Costs and outcomes were used to calculate the incremental cost-effectiveness ratio (ICER) of M-CT relative to TAU alone [40]. The following formula was used for calculating the ICER:

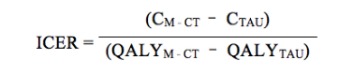



...where C_M-CT_ and C_TAU_ are the mean costs, and QALY_M-CT_ and QALY_TAU_ are the mean QALYs in M-CT and TAU, respectively. The ICER is interpreted as the additional costs per QALY gained when M-CT is offered rather than TAU. The bootstrap method [41] was applied to account for the uncertainty in the economic evaluation by repeated random sampling with replacement from the original dataset. Seemingly unrelated regression equations were bootstrapped (5000 times) to allow for correlated residuals of the cost and utility equations and adjust for baseline differences in one of the sensitivity analyses. In each bootstrap step, the mean cost differences and the mean outcome differences were computed and these were plotted in the cost-effectiveness plane [42]. Finally, cost-effectiveness acceptability curves (CEACs) [43] were graphed, taking into account the relative placement of the bootstrap replications. CEACs inform decision makers on the likelihood that an intervention is deemed to be cost effective given a range of willingness-to-pay ceilings for gaining an additional unit of health (ie, gaining one QALY and gaining one depression-free day). The analyses were conducted using Stata (StataCorp LLC).

### Sensitivity Analyses

Due to the amount of missing data, we used multiple imputations in the main analysis to handle missing data. To ascertain the robustness of our findings, we performed several sensitivity analyses, each handling missing data in a different way. It should be noted that in the main analysis, 29 participants were not included since they dropped out immediately after randomization and no follow-up information was available. Nevertheless, we performed an additional sensitivity analysis in which all participants were included for a full-fledged intention-to-treat analysis. The main analysis was repeated again but now restricted to individuals for whom at least 50% of the cost data were available. A final analysis to evaluate the impact of drop-out was based on complete cases. At baseline, we observed a slight imbalance between both conditions with respect to gender, severity of the last MDE, and baseline costs. Studies suggest that gender is not associated with relapse or recurrence but that severity of the last MDE might be [25]. Therefore, we repeated the main analysis now adjusting for the small baseline imbalances in severity of the last MDE and baseline costs.

## Results

### Sample Characteristics

The participant flow during the study is displayed in Multimedia Appendix 3. In total, 288 participants were eligible of whom 264 were randomized to either M-CT added to TAU (n=132) or TAU alone (n=132). In total, 29 participants dropped out immediately after randomization and 24 were lost to follow-up during the study. Overall, the baseline clinical and demographic characteristics of all participants (Table 1) and participants with any follow-up data were similar and equally distributed over the treatment conditions, suggesting no systematic bias due to drop-out of the 29 individuals with no follow-up data. Complete cost and effect data (available for all measurements) were available for 19.1 % (45/235) participants. At least one measurement of cost data after baseline was available for 83.0% (195/235) of the participants. For 54.9% (129/235) of the participants, at least half of the cost measurements were available.

**Table 1:** Sample characteristics by condition at baseline. Values may not add up to 100% because of missing data.

Characteristics		M-CT^a^ (n=132)	TAU^b^ (n=132)
Age (years), mean (SD)		45.6 (10.9)	47.1 (10.7)
Female, n (%)		105 (79.5)	92 (69.7)
Country of birth, The Netherlands, n (%)		116 (88.5)	121 (92.4)
**Marital status, n** **(%)**			
	Single	39 (29.8)	32 (24.2)
	Married or cohabiting	82 (62.6)	87 (65.9)
	Divorced/widowed	10 (7.7)	13 (9.9)
**Education, n (%)**			
	Primary and/or secondary education	17 (12.9)	22 (16.7)
	Vocational education	30 (22.7)	34 (25.8)
	Higher education	85 (64.4)	76 (57.6)
Employed, n (%)		87 (66.4)	90 (68.7)
**Treatment as usual, n** **(%)**			
	No treatment	46 (34.8)	39 (30.0)
	General practitioner	34 (25.8)	43 (33.1)
	Specialized mental health aftercare	52 (39.4)	48 (36.9)
Treatment with antidepressants, n (%)		72 (55.4)	65 (50.8)
Previous episodes MDD^c^, median (IQR^d^)		4 (2.8)	4 (2.0)
Total HRSD^e^_,_ mean (SD)		3.7 (3.1)	3.4 (2.9)
Depressive symptoms (IDS-SR^f^), mean (SD)		16.5 (10.3)	16.3 (9.7)
**Severity past episode, n** **(%)**			
	Mild	37 (28.0)	25 (18.9)
	Moderate	73 (55.3)	71 (53.8)
	Severe	22 (16.7)	36 (27.3)
Baseline utilities (EQ-5D-3L^g^), mean (SD)		0.86 (0.12)	0.84 (0.17)
Baseline costs (€), mean (SD)		1729 (3699)	1552 (3216)

^a^M-CT: mobile cognitive therapy.

^b^TAU: treatment as usual.

^c^MDD: major depressive disorder.

^d^IQR: interquartile range.

^e^HRSD: Hamilton Rating Scale for Depression.

^f^IDS-SR: Inventory of Depressive Symptomatology, Self-Report.

^g^EQ-5D-3L: three level version of the EuroQol Five Dimensional Questionnaire.

### Costs

The various types of costs generated by both groups during the 24 months of the study are presented in Multimedia Appendix 4. Presented costs were based on the data of participants for whom at least one cost measurement after the baseline assessment was available during the study. Mean costs of the M-CT intervention were €73 (US $83) per participant. These costs were mainly related to the training and supervision of therapists, contacts between therapists and participants, and information and communication technology costs (development and periodically upgrading the software and server costs). In both conditions, the costs of hospital admissions and outpatient care provided by mental health care services contributed considerably to the overall costs within the health care sector. Costs related to productivity losses had the largest impact on overall societal costs. Mean total costs accrued over 24 months were €8298 (US $9410) for M-CT and €7296 (US $8274) for TAU.

### Outcomes

The mean number of depression-free days within the 24 months of the study was 661 in M-CT and 656 in TAU. Mean QALYs were 1.65 for both M-CT and TAU. No statistically significant differences in depression-free days and QALYs were found (*t*=0.43, *P*=.67 and *t*=0.18, *P*=.86, respectively).

### Economic Evaluation

**Figure 1:**
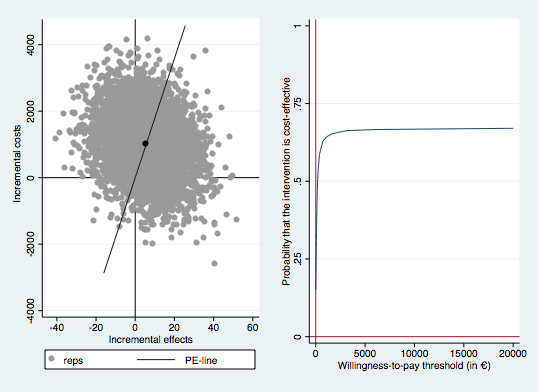
Incremental cost-effectiveness plane (left) and cost-effectiveness acceptability curve (right) for the 5000 bootstrapped incremental costs per depression-free day gained. Reps: bootstrap replication; PE-line: line which represents the point estimate of the ICER (average cost/effect of bootstrap replications).

**Figure 2:**
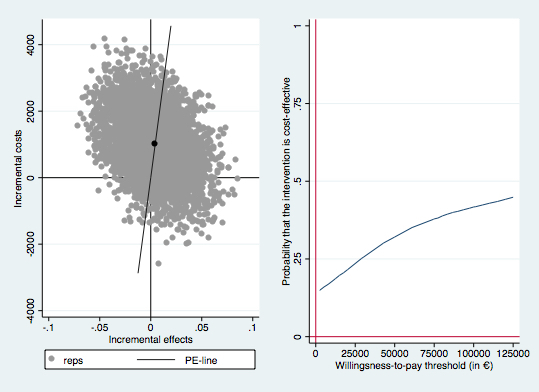
Incremental cost-effectiveness plane (left) and cost-effectiveness acceptability curve (right) for the 5000 bootstrapped incremental costs per quality-adjusted life years gained. Reps: bootstrap replication; PE-line: line which represents the point estimate of the ICER (average cost/effect of bootstrap replications).

According to the main analysis, M-CT resulted in an extra 5.6 gain in depression-free days (95% CI 5.3-6.0) and a 0.004 QALY gain (95% CI 0.004-0.005)—but these health gains were achieved at higher costs (€1008 [US $1143], 95% CI €983-€1034). For both health outcomes, most of the bootstrapped ICERs were located in the northeast quadrant (55.5% for depression-free days and 46.5% for QALYs), indicating that the probability that M-CT is deemed cost effective depends on the willingness-to-pay for an additional health gain (see Figures 1 and 2). When the willingness-to-pay per additional depression-free day is zero, M-CT has approximately a 40% probability of being cost effective. When the willingness-to-pay per additional gain in depression-free days increases, the probability that M-CT is cost effective also increases but does not rise above 65%. For QALYs, increased willingness-to-pay only leads to slight increases in the probability that M-CT will be considered cost effective and the probability that M-CT is cost effective does not rise above 40%.

### Sensitivity Analyses

Table 2 displays the main analysis and sensitivity analyses. The sensitivity analysis including all randomized participants (n=264) overall yielded similar results. When taking into account participants for whom at least 50% of the data were available, TAU dominated M-CT in terms of depression-free days, and results were roughly similar to the main analysis for QALYs. In the complete case analysis, TAU dominated M-CT. Adjustments for imbalanced baseline variables yielded similar results.

**Table 2:** Sensitivity analyses with all estimates based on 5000 bootstrap replications.

Characteristics		Costs and effects				Mean ICER^a^ (€)		Distribution of cost- effectiveness plane					
		Incremental costs (€)		Incremental effects				NE^b^ (%)		SE^c^ (%, dominant)		SW^d^ (%)	NW^e^ (%, inferior)
**Cost effectiveness, depression-free days**													
	Main analysis^f^ (n=235)		1008 (983-1034)		5.6 (5.3 to 6.0)		179		55.5		11.6	2.6	30.2
	Main analysis all participants^g^ (n=264)		1033 (1000-1065)		4.7 (4.4 to 5.0)		140		51.7		15.5	3.8	29.1
	≥50% follow-up data (n=129)		1562 (1508-1617)		–34.7 (–35.2 to –34.1)		(dominated)		1.8		1.3	20.3	76.6
	Complete case (n=45)		2086 (1990-2181)		–51.7 (–52.5 to –50.9)		(dominated)		1.8		1.3	20.3	76.6
**Cost utility, QALYs^h^**													
	Main analysis (n=235)		1008 (983-1034)		0.004 (0.004 to 0.005)		230,816		46.5		11.2	3.0	39.3
	Main analysis all participants (n=264)		1033 1000-1065)		0.02 (0.02 to 0.02)		53,583		63.9		16.5	1.6	18.1
	≥50% follow-up data (n=129)		1562 (1508-1617)		0.02 (0.02 to (0.02)		87,676		48.8		20.0	2.6	29.6
	Complete case (n=45)		2086 (1990-2181)		–0.04 (–0.05 to –0.04)		(dominated)		15.6		11.9	15.7	56.8

^a^ICER: incremental cost-effectiveness ratio.

^b^NE: northeast.

^c^SE: southeast.

^d^SW: southwest.

^e^NW: northwest.

^f^Main analysis using multiple imputations with predictive mean matching, excluding the 29 participants with no follow-up data.

^g^Main analysis but now with all participants including the 29 participants without follow-up data.

^h^QALY: quality-adjusted life year.

## Discussion

### Principal Findings and Comparison With Prior Work

This study was the first to evaluate the cost effectiveness and cost utility of an internet-based relapse prevention program for recurrent MDD. The results of this study suggest that M-CT added to TAU is not cost effective compared to TAU alone over a 24-month period.

The results revealed that the total costs in both conditions were dominated by losses in productivity, which is consistent with Krol et al [44] and Biesheuvel-Leliefeld et al (unpublished data, 2018). Krol et al [44] found that choices made regarding the inclusion or exclusion of indirect costs influence cost-effectiveness studies to a great extent. As MDD is a disorder with extensive losses in worker productivity [4-7,44], we believe that the societal perspective, in which both direct and indirect costs are included, is justified. Our main analysis showed that participants using M-CT had slightly more depression-free days (5.6 days) and better QALYs (0.004) compared to TAU but achieved at higher costs (€1008 [US $1143]). The probability that M-CT was cost effective in terms of depression-free days and QALYs was dependent on the willingness-to-pay for an additional health gain. For depression-free days, a substantial investment had to be made before reaching an acceptable probability that M-CT was deemed cost effective. For QALYs, a substantial investment had to be made but the probability that M-CT was cost effective did not rise above 40%. We conclude that adding M-CT to TAU is not a cost-effective strategy compared to TAU alone. The results of the sensitivity analyses were partly inconsistent with the main analysis and will be further discussed.

The results contrast with our expectations based on the positive short-term results of M-CT [45] and with the promising findings of Holländare [18] regarding the internet-based relapse prevention therapy in partially remitted individuals. Moreover, the results contrast with the long-term effectiveness of face-to-face PCT that is found by Bockting et al [27-29,46] and De Jonge et al (unpublished data, 2018) and with PCT administered as bibliotherapy with therapist support [47]. As reported elsewhere [20], treatment adherence in this study was comparable to other studies (68.2% [90/132] finished at least 5 lessons) [15] and, therefore, we believe this did not explain the results. Our short-term clinical results demonstrated a positive effect of M-CT on residual depressive symptoms during the first months compared to TAU [45], whereas our long-term results showed no protective effects of M-CT on cumulative relapse/recurrence rate, number of depressive relapses/recurrences, and depressive symptoms after 24 months [20]. Therefore, we hypothesize that M-CT might generate better effects at lower costs during the first months and hence might be more cost effective compared to TAU but that the costs and effects become less favorable for M-CT in the long run. This hypothesis is consistent with the meta-analytic review of So et al [48] that found short-term effects of internet-based treatments for MDD but no effects beyond 6 months posttreatment. However, a recent review on the long-term effects of internet-based guided cognitive behavioral therapy showed enduring effects for several disorders [49]. We hypothesize that more therapist support and/or booster sessions are needed for M-CT to become more cost effective in the long term. In this study, minimal therapist support was enlisted by the participants (ie, a mean total therapist time of only 17.3 minutes [range 0-70] per participant). Several systematic reviews and meta-analyses showed that internet-based interventions are especially effective when provided with therapist support [13,15,16]. Therapist guidance also seems an important factor in economic evaluations of internet-based interventions. A recent systematic review on economic evaluations of internet- and mobile-based interventions for the treatment and prevention of depression concluded that the internet-based interventions that were likely to be cost effective were all guided interventions whereas the unguided interventions were not likely to be cost effective or the results were ambiguous [50]. However, it is important to note that for most of these guided interventions, a considerable investment was needed in order to reach an acceptable probability that the intervention was deemed cost effective. In their systematic review on the cost effectiveness of internet-based interventions for a wide range of mental health disorders, Donker et al [51] highlighted that the most robust evidence in terms of cost effectiveness was found for guided interventions. A recent individual-participant data meta-analysis on the cost effectiveness of guided internet-based interventions for depression based on 5 studies concluded that considerable investments had to be made for an acceptable probability that the intervention would be cost effective compared to controls in terms of treatment response and depressive symptoms and that for QALYs, this probability was low at the widely used willingness-to-pay-threshold [52]. Differences between the systematic reviews and meta-analysis might be caused by differences in methodology, control group, and/or differences in when an intervention is perceived cost effective. For example, in the systematic review of Paganini et al [50], studies examining only direct health care costs were also included and 4 out of the 7 studies classified as cost effective used a wait list control group as comparator, whereas in the meta-analysis of Kolovos et al [52], only studies that also took into account productivity losses were included and only one study included a wait list control group.

Thus, more information is needed under which circumstances internet-based treatments are effective and cost effective regarding short-term and long-term outcomes. Based on their systematic review, Erbe et al [53] concluded that combining the strength of both face-to-face and internet-based interventions might be a promising direction, although more studies are needed. In addition, besides examining the (cost) effectiveness of specific internet-based interventions, future studies should focus more on the implementation in clinical practice, taking into account specific barriers (eg, preferences of individuals and professionals) [54,55]. Furthermore, more information is needed under which circumstances face-to-face or other forms of PCT are cost effective. To date, one study examined the cost effectiveness of PCT administered as guided bibliotherapy for remitted recurrently depressed individuals (Biesheuvel-Leliefeld et al, unpublished data, 2018) and another study examined the cost effectiveness of PCT in remitted recurrently depressed individuals that had received acute-phase cognitive therapy (De Jonge et al, unpublished data, 2018). Both studies concluded that investments had to be made for an acceptable probability that the intervention would be cost effective in terms of recurrences. For QALYs, substantial investments had to be made but probabilities that the intervention would be cost effective remained low.

### Limitations

Some limitations of this study are important to acknowledge. First, cost data were collected with a self-report questionnaire approximately every 3 months during the 24-month follow-up and therefore a substantial amount of participants missed one or more measurement. To deal with missing data, multiple imputations, which is a recommended strategy to handle missing data in cost-effectiveness studies performed alongside RCTs [56,57], were used in our main analysis. We can assume the data were at least partly missing at random since baseline characteristics predicted whether the data were missing. Nevertheless, the missing completely at random assumption cannot be proved, and it is possible data were missing not at random because drop-out could be related to depressive symptom severity. Because of the amount of missing data, we did not want to rely on a single imputation technique and therefore performed several sensitivity analyses that each handled missing data in a different way. The main analysis, the analysis including all participants, and the analysis incorporating participants for whom at least 50% of the data were available (the latter only regarding QALYs) showed similar results. The analysis including participants with at least 50% data (regarding depression-free days) and complete cases showed higher costs and worse outcomes for M-CT compared to TAU. Multiple imputations are preferred over a complete case analysis because of the potential selection bias that might occur due to missing values. The results of the complete cases and cases with at least 50% of the data do suggest a possible selection bias in drop-out, which is also suggested when inspecting a baseline table displaying only the complete cases and cases with 50% of the data. Altogether, we regard our main analysis as primary. Second, the data were obtained in the Netherlands, and generalizability into other countries with other treatment settings is questionable. Third, the cost data and data for the cost-utility analysis were based on retrospective self-report questionnaires which may have affected the reliability. The TiC-P has shown to be a reliable and valid questionnaire for collecting cost data [58]. However, the EQ-5D might be subjected to a possible ceiling effect when estimating changes in QALYs for this group of remitted individuals. Moreover, the EQ-5D refers to the current health state and therefore does not capture all relapses/recurrences during the 24 months of the study.

### Conclusions

Although the effectiveness of internet-based therapy for depression is currently established, only a few studies examined the cost effectiveness of these interventions [17,50-52,59,60]. We conclude that adding M-CT to TAU is not an effective or cost-effective strategy to prevent relapse and recurrence. Future studies should examine the long-term effectiveness of internet-based interventions and the optimal dosage of guidance by therapists. MDD is highly recurrent [2] and one of the leading causes of disability [1,3]. Therefore, it is important that future studies continue to examine highly accessible, scalable, and (potentially) cost-effective interventions to treat depression including interventions that prevent relapse and recurrence. These studies are needed to inform decisions in mental health care. Since treatment effects can manifest differently over time [48], it is important that these cost-effectiveness studies on face-to-face and internet-based interventions include long follow-up periods.

## Appendix

Multimedia Appendix 1 CONSORT‐EHEALTH checklist (V 1.6.1).

Multimedia Appendix 2 Consolidated Health Economic Evaluation Reporting Standards statement.

Multimedia Appendix 3 Consolidated Standards of Reporting Trials flow diagram over the 24-month follow-up.

Multimedia Appendix 4 Cumulative costs (€) inside and outside the health care sector over 24 months.

## Abbreviations

CEAC: cost-effectiveness acceptability curve
DSM-IV: Diagnostic and Statistical Manual of Mental Disorders, Fourth Edition
EQ-5D-3L: three level version of the EuroQol Five Dimensional Questionnaire.
HRSD: Hamilton Rating Scale for Depression
ICER: incremental cost-effectiveness ratio
M-CT: mobile cognitive therapy
MDD: major depressive disorder
MDE: major depressive episode
PCT: preventive cognitive therapy
QALY: quality-adjusted life year
RCT: randomized controlled trial
SCID: Structured Clinical Interview for DSM Disorders
TAU: treatment as usual
TiC-P: Trimbos and Institute for Medical Technology Assessment Questionnaire on Costs Associated with Psychiatric Illness
