# Supplementary material for: Economic Evaluation of an Internet-Based Preventive Cognitive Therapy With Minimal Therapist Support for Recurrent Depression: Randomized Controlled Trial
Source: J Med Internet Res. 2018 Nov 26;20(11):e10437. doi: 10.2196/10437 (PMC6288594; doi:10.2196/10437)
Supplement: Multimedia Appendix 1 [file jmir_v20i11e10437_app1.pdf]

CONSORT-EHEALTH (V 1.6.1) - Submission/Publication Form

The CONSORT-EHEALTH checklist is intended for authors of randomized trials evaluating web-based and Internet-based applications/interventions, including mobile interventions, electronic games (incl multiplayer games), social media, certain telehealth applications, and other interactive and/or networked electronic applications. Some of the items (e.g. all subitems under item 5 - description of the intervention) may also be applicable for other study designs.

The goal of the CONSORT EHEALTH checklist and guideline is to be
a) a guide for reporting for authors of RCTs,
b) to form a basis for appraisal of an ehealth trial (in terms of validity)

CONSORT-EHEALTH items/subitems are MANDATORY reporting items for studies published in the Journal of Medical Internet Research and other journals / scientific societies endorsing the checklist.

Items numbered 1., 2., 3., 4a., 4b etc are original CONSORT or CONSORT-NPT (non-pharmacologic treatment) items.
Items with Roman numerals (i., ii, iii, iv etc.) are CONSORT-EHEALTH extensions/clarifications.

As the CONSORT-EHEALTH checklist is still considered in a formative stage, we would ask that you also RATE ON A SCALE OF 1-5 how important/useful you feel each item is FOR THE PURPOSE OF THE CHECKLIST and reporting guideline (optional).

Mandatory reporting items are marked with a red *.
In the textboxes, either copy & paste the relevant sections from your manuscript into this form - please include any quotes from your manuscript in QUOTATION MARKS,
or answer directly by providing additional information not in the manuscript, or elaborating on why the item was not relevant for this study.

YOUR ANSWERS WILL BE PUBLISHED AS A SUPPLEMENTARY FILE TO YOUR PUBLICATION IN JMIR AND ARE CONSIDERED PART OF YOUR PUBLICATION (IF ACCEPTED).
Please fill in these questions diligently. Information will not be copyedited, so please use proper spelling and grammar, use correct capitalization, and avoid abbreviations.

DO NOT FORGET TO SAVE AS PDF _AND_ CLICK THE SUBMIT BUTTON SO YOUR ANSWERS ARE IN OUR DATABASE !!!

Citation Suggestion (if you append the pdf as Appendix we suggest to cite this paper in the caption):
Eysenbach G, CONSORT-EHEALTH Group
CONSORT-EHEALTH: Improving and Standardizing Evaluation Reports of Web-based and Mobile Health Interventions
J Med Internet Res 2011;13(4):e126
URL: http://www.jmir.org/2011/4/e126/
[illegible]

You're editing your response. Sharing this URL allows others to also edit your response.

FILL IN A NEW RESPONSE

Your name *

First Last

Nicola Klein

Primary Affiliation (short), City, Country *

University of Toronto, Toronto, Canada

University of Groningen, Groningen, The N

Your e-mail address *

abc@gmail.com

n.s.klein@rug.nl

Title of your manuscript *

Provide the (draft) title of your manuscript.

Economic evaluation of an internet-based preventive cognitive therapy with minimal therapist support for recurrent depression: results of a randomized controlled trial

Name of your App/Software/Intervention *

If there is a short and a long/alternate name, write the short name first and add the long name in brackets.

M-CT (Mobile Cognitive Therapy)

Evaluated Version (if any)

e.g. "V1", "Release 2017-03-01", "Version 2.0.27913"

Your answer

You're editing your response. Sharing this URL allows others to also edit your response.

FILL IN A NEW RESPONSE

Language(s) *

What language is the intervention/app in? If multiple languages are available, separate by comma (e.g. "English, French")

Dutch

URL of your Intervention Website or App

e.g. a direct link to the mobile app on app in appstore (itunes, Google Play), or URL of the website. If the intervention is a DVD or hardware, you can also link to an Amazon page.

https://www.depressievrij.nl/

URL of an image/screenshot (optional)

Your answer

Accessibility *

Can an enduser access the intervention presently?

- ○ access is free and open
- ○ access only for special usergroups, not open
- ○ access is open to everyone, but requires payment/subscription/in-app purchases
- ◉ app/intervention no longer accessible
- ○ Other:

Primary Medical Indication/Disease/Condition *

e.g. "Stress", "Diabetes", or define the target group in brackets after the condition, e.g. "Autism (Parents of children with)", "Alzheimers (Informal Caregivers of)"

Remitted recurrent depression

You're editing your response. Sharing this URL allows others to also edit your response.

FILL IN A NEW RESPONSE

Primary Outcomes measured in trial *

comma-separated list of primary outcomes reported in the trial

Depression-free days, QALYs

Secondary/other outcomes

Are there any other outcomes the intervention is expected to affect?

N/A

Recommended "Dose" *

What do the instructions for users say on how often the app should be used?

- ( ) Approximately Daily
- (•) Approximately Weekly
- ( ) Approximately Monthly
- ( ) Approximately Yearly
- ( ) "as needed"
- ( ) Other:

You're editing your response. Sharing this URL allows others to also edit your response.

FILL IN A NEW RESPONSE

Approx. Percentage of Users (starters) still using the app as recommended after 3 months *

- ○ unknown / not evaluated
- ○ 0-10%
- ○ 11-20%
- ○ 21-30%
- ○ 31-40%
- ○ 41-50%
- ○ 51-60%
- ◉ 61-70%
- ○ 71%-80%
- ○ 81-90%
- ○ 91-100%
- ○ Other:

Overall, was the app/intervention effective? *

- ○ yes: all primary outcomes were significantly better in intervention group vs control
- ○ partly: SOME primary outcomes were significantly better in intervention group vs control
- ◉ no statistically significant difference between control and intervention
- ○ potentially harmful: control was significantly better than intervention in one or more outcomes
- ○ inconclusive: more research is needed

You're editing your response. Sharing this URL allows others to also edit your response.

FILL IN A NEW RESPONSE

Article Preparation Status/Stage *

At which stage in your article preparation are you currently (at the time you fill in this form)

- ○ not submitted yet - in early draft status
- ○ not submitted yet - in late draft status, just before submission
- ○ submitted to a journal but not reviewed yet
- ◉ submitted to a journal and after receiving initial reviewer comments
- ○ submitted to a journal and accepted, but not published yet
- ○ published
- ○ Other:

Journal *

If you already know where you will submit this paper (or if it is already submitted), please provide the journal name (if it is not JMIR, provide the journal name under "other")

- ○ not submitted yet / unclear where I will submit this
- ◉ Journal of Medical Internet Research (JMIR)
- ○ JMIR mHealth and UHealth
- ○ JMIR Serious Games
- ○ JMIR Mental Health
- ○ JMIR Public Health
- ○ JMIR Formative Research
- ○ Other JMIR sister journal
- ○ Other:

Is this a full powered effectiveness trial or a pilot/feasibility trial? *

You're editing your response. Sharing this URL allows others to also edit your response.

FILL IN A NEW RESPONSE

Manuscript tracking number *

If this is a JMIR submission, please provide the manuscript tracking number under "other" (The ms tracking number can be found in the submission acknowledgement email, or when you login as author in JMIR. If the paper is already published in JMIR, then the ms tracking number is the four-digit number at the end of the DOI, to be found at the bottom of each published article in JMIR)

- ○ no ms number (yet) / not (yet) submitted to / published in JMIR
- ◉ Other: ms#10437

TITLE AND ABSTRACT

1a) TITLE: Identification as a randomized trial in the title

1a) Does your paper address CONSORT item 1a? *

I.e does the title contain the phrase "Randomized Controlled Trial"? (if not, explain the reason under "other")

- ◉ yes
- ○ Other:

1a-i) Identify the mode of delivery in the title

Identify the mode of delivery. Preferably use “web-based” and/or “mobile” and/or “electronic game” in the title. Avoid ambiguous terms like “online”, “virtual”, “interactive”. Use “Internet-based” only if Intervention includes non-web-based Internet components (e.g. email), use “computer-based” or “electronic” only if offline products are used. Use “virtual” only in the context of “virtual reality” (3-D worlds). Use “online” only in the context of “online support groups”. Complement or substitute product names with broader terms for the class of products (such as “mobile” or “smart phone” instead of “iphone”), especially if the application runs on different platforms.

	1	2	3	4	5	
subitem not at all important	○	○	○	○	○	essential

You're editing your response. Sharing this URL allows others to also edit your response.

FILL IN A NEW RESPONSE

Does your paper address subitem 1a-i? *

Copy and paste relevant sections from manuscript title (include quotes in quotation marks "like this" to indicate direct quotes from your manuscript), or elaborate on this item by providing additional information not in the ms, or briefly explain why the item is not applicable/relevant for your study

"internet-based preventive cognitive therapy"

1a-ii) Non-web-based components or important co-interventions in title

Mention non-web-based components or important co-interventions in title, if any (e.g., “with telephone support”).

	1	2	3	4	5	
subitem not at all important	○	○	○	○	○	essential

Does your paper address subitem 1a-ii?

Copy and paste relevant sections from manuscript title (include quotes in quotation marks "like this" to indicate direct quotes from your manuscript), or elaborate on this item by providing additional information not in the ms, or briefly explain why the item is not applicable/relevant for your study

"with minimal therapist support"

1a-iii) Primary condition or target group in the title

Mention primary condition or target group in the title, if any (e.g., “for children with Type I Diabetes”) Example: A Web-based and Mobile Intervention with Telephone Support for Children with Type I Diabetes: Randomized Controlled Trial

	1	2	3	4	5	
subitem not at all important	○	○	○	○	○	essential

Does your paper address subitem 1a-iii? *

Copy and paste relevant sections from manuscript title (include quotes in quotation marks "like this" to indicate direct quotes from your manuscript), or elaborate on this item by providing additional information not in the ms, or briefly explain why the item is not applicable/relevant for your study

"for recurrent depression"

You're editing your response. Sharing this URL allows others to also edit your response.

FILL IN A NEW RESPONSE

1b) ABSTRACT: Structured summary of trial design, methods, results, and conclusions

NPT extension: Description of experimental treatment, comparator, care providers, centers, and blinding status.

1b-i) Key features/functionalities/components of the intervention and comparator in the METHODS section of the ABSTRACT

Mention key features/functionalities/components of the intervention and comparator in the abstract. If possible, also mention theories and principles used for designing the site. Keep in mind the needs of systematic reviewers and indexers by including important synonyms. (Note: Only report in the abstract what the main paper is reporting. If this information is missing from the main body of text, consider adding it)

	1	2	3	4	5	
subitem not at all important	○	○	○	○	○	essential

Does your paper address subitem 1b-i? *

Copy and paste relevant sections from the manuscript abstract (include quotes in quotation marks "like this" to indicate direct quotes from your manuscript), or elaborate on this item by providing additional information not in the ms, or briefly explain why the item is not applicable/relevant for your study

"In total, 288 remitted individuals with a history of recurrent depression were eligible, of whom 264 were randomly allocated to M-CT with minimal therapist support added to Treatment As Usual (TAU) or TAU alone. M-CT comprised eight online lessons and participants were advised to complete one lesson per week."

1b-ii) Level of human involvement in the METHODS section of the ABSTRACT

Clarify the level of human involvement in the abstract, e.g., use phrases like “fully automated” vs. “therapist/nurse/care provider/physician-assisted” (mention number and expertise of providers involved, if any). (Note: Only report in the abstract what the main paper is reporting. If this information is missing from the main body of text, consider adding it)

	1	2	3	4	5	
subitem not at [illegible]	○	○	○	○	○	essential

You're editing your response. Sharing this URL allows others to also edit your response.

FILL IN A NEW RESPONSE

Does your paper address subitem 1b-ii?

Copy and paste relevant sections from the manuscript abstract (include quotes in quotation marks "like this" to indicate direct quotes from your manuscript), or elaborate on this item by providing additional information not in the ms, or briefly explain why the item is not applicable/relevant for your study

"with minimal therapist support + The participants scarcely booked additional therapist support, resulting in 17.3 minutes of mean total therapist support."

1b-iii) Open vs. closed, web-based (self-assessment) vs. face-to-face assessments in the METHODS section of the ABSTRACT

Mention how participants were recruited (online vs. offline), e.g., from an open access website or from a clinic or a closed online user group (closed usergroup trial), and clarify if this was a purely web-based trial, or there were face-to-face components (as part of the intervention or for assessment). Clearly say if outcomes were self-assessed through questionnaires (as common in web-based trials). Note: In traditional offline trials, an open trial (open-label trial) is a type of clinical trial in which both the researchers and participants know which treatment is being administered. To avoid confusion, use “blinded” or “unblinded” to indicated the level of blinding instead of “open”, as “open” in web-based trials usually refers to “open access” (i.e. participants can self-enrol). (Note: Only report in the abstract what the main paper is reporting. If this information is missing from the main body of text, consider adding it)

	1	2	3	4	5	
subitem not at all important	○	○	○	○	○	essential

Does your paper address subitem 1b-iii?

Copy and paste relevant sections from the manuscript abstract (include quotes in quotation marks "like this" to indicate direct quotes from your manuscript), or elaborate on this item by providing additional information not in the ms, or briefly explain why the item is not applicable/relevant for your study

"Participants were recruited via media, general practitioners, and mental health care institutions."

1b-iv) RESULTS section in abstract must contain use data

Report number of participants enrolled/assessed in each group, the use/uptake of the intervention (e.g., attrition/adherence metrics, use over time, number of logins etc.), in addition to primary/secondary outcomes. (Note: Only report in the abstract what the main paper is reporting. If this information is missing from the main body of text, consider adding it)

	1	2	3	4	5	
subitem not at all important	○	○	○	○	○	essential

You're editing your response. Sharing this URL allows others to also edit your response.

FILL IN A NEW RESPONSE

Does your paper address subitem 1b-iv?

Copy and paste relevant sections from the manuscript abstract (include quotes in quotation marks "like this" to indicate direct quotes from your manuscript), or elaborate on this item by providing additional information not in the ms, or briefly explain why the item is not applicable/relevant for your study

"In total, 288 remitted individuals with a history of recurrent depression were eligible, of whom 264 were randomly allocated + Adherence rates were similar to other studies and therefore do not explain this finding. The participants scarcely booked additional therapist support, resulting in 17.3 minutes of mean total therapist support."

1b-v) CONCLUSIONS/DISCUSSION in abstract for negative trials

Conclusions/Discussions in abstract for negative trials: Discuss the primary outcome - if the trial is negative (primary outcome not changed), and the intervention was not used, discuss whether negative results are attributable to lack of uptake and discuss reasons. (Note: Only report in the abstract what the main paper is reporting. If this information is missing from the main body of text, consider adding it)

	1	2	3	4	5	
subitem not at all important	○	○	○	○	○	essential

Does your paper address subitem 1b-v?

Copy and paste relevant sections from the manuscript abstract (include quotes in quotation marks "like this" to indicate direct quotes from your manuscript), or elaborate on this item by providing additional information not in the ms, or briefly explain why the item is not applicable/relevant for your study

"Adherence rates were similar to other studies and therefore do not explain this finding. The participants scarcely booked additional therapist support, resulting in 17.3 minutes of mean total therapist support. More studies are needed to examine the cost-effectiveness of internet-based interventions with respect to long-term outcomes and the role and optimal dosage of therapist support. Overall, more research is needed on scalable and cost-effective interventions that can reduce the burden of recurrent MDD."

INTRODUCTION

2a) In INTRODUCTION: Scientific background and explanation of

You're editing your response. Sharing this URL allows others to also edit your response.

FILL IN A NEW RESPONSE

2a-i) Problem and the type of system/solution

Describe the problem and the type of system/solution that is object of the study: intended as stand-alone intervention vs. incorporated in broader health care program? Intended for a particular patient population? Goals of the intervention, e.g., being more cost-effective to other interventions, replace or complement other solutions? (Note: Details about the intervention are provided in "Methods" under 5)

	1	2	3	4	5	
subitem not at all important	○	○	○	○	○	essential

You're editing your response. Sharing this URL allows others to also edit your response.

FILL IN A NEW RESPONSE

Does your paper address subitem 2a-i? *

Copy and paste relevant sections from the manuscript (include quotes in quotation marks "like this" to indicate direct quotes from your manuscript), or elaborate on this item by providing additional information not in the ms, or briefly explain why the item is not applicable/relevant for your study

"To alleviate the burden of MDD, psychological interventions and/or antidepressants (ADs) are recommended for the acute phase of MDD and further as continuation/maintenance therapy to prevent relapse and recurrence [8]. However, health care resources are limited and many individuals fail to seek treatment [9-12]. Because of their flexible and accessible nature, internet-based interventions could be a viable cost-effective solution that reaches a large number of ‘at risk’ individuals. The effectiveness of internet-based interventions in acute and residual MDD has been established [13-17], with small to moderate effect sizes for interventions without therapist support and higher effect sizes with therapist support [e.g., 13,15]. To date, only one study examined the long-term effects of an internet-based relapse prevention program [18] and no study examined its cost-effectiveness. Only a single study aimed at the prevention of MDD examined the cost-effectiveness of an internet-based relapse prevention program but this study aimed to prevent the first onset of MDD [19]. Thus, little is known about the cost-effectiveness of internet-based relapse prevention for recurrent MDD. More information is needed on the health impact and economic costs to inform policy makers and health care providers.
In our Randomized Controlled Trial (RCT), we examined the clinical effectiveness of an internet-based relapse prevention program (Mobile Cognitive Therapy, M-CT) added to Treatment As Usual (TAU) compared to TAU alone in remitted individuals with recurrent MDD. Results showed that M-CT added to TAU was slightly but not significantly superior to TAU alone after 24 months in terms of cumulative relapse/recurrence rate, number of depressive relapses, and depressive symptoms [20]. In the current study, we evaluated the cost-effectiveness and cost-utility of M-CT to see if the economic case could be made for this low cost and highly scalable intervention that was added to TAU. "

You're editing your response. Sharing this URL allows others to also edit your response.

FILL IN A NEW RESPONSE

2a-ii) Scientific background, rationale: What is known about the (type of) system

Scientific background, rationale: What is known about the (type of) system that is the object of the study (be sure to discuss the use of similar systems for other conditions/diagnoses, if appropiate), motivation for the study, i.e. what are the reasons for and what is the context for this specific study, from which stakeholder viewpoint is the study performed, potential impact of findings [2]. Briefly justify the choice of the comparator.

	1	2	3	4	5	
subitem not at all important	○	○	○	○	○	essential

Does your paper address subitem 2a-ii? *

Copy and paste relevant sections from the manuscript (include quotes in quotation marks "like this" to indicate direct quotes from your manuscript), or elaborate on this item by providing additional information not in the ms, or briefly explain why the item is not applicable/relevant for your study

"Because of their flexible and accessible nature, internet-based interventions could be a viable cost-effective solution that reaches a large number of ‘at risk’ individuals. The effectiveness of internet-based interventions in acute and residual MDD has been established [13-17], with small to moderate effect sizes for interventions without therapist support and higher effect sizes with therapist support [e.g., 13,15]."

2b) In INTRODUCTION: Specific objectives or hypotheses

Does your paper address CONSORT subitem 2b? *

Copy and paste relevant sections from the manuscript (include quotes in quotation marks "like this" to indicate direct quotes from your manuscript), or elaborate on this item by providing additional information not in the ms, or briefly explain why the item is not applicable/relevant for your study

"In the current study, we evaluated the cost-effectiveness and cost-utility of M-CT to see if the economic case could be made for this low cost and highly scalable intervention that was added to TAU. We hypothesize that M-CT added to TAU is cost-effective compared to TAU alone as it might generate slightly better health outcomes and thereby lower costs for other mental health services."

You're editing your response. Sharing this URL allows others to also edit your response.

FILL IN A NEW RESPONSE

3a) Description of trial design (such as parallel, factorial) including allocation ratio

Does your paper address CONSORT subitem 3a? *

Copy and paste relevant sections from the manuscript (include quotes in quotation marks "like this" to indicate direct quotes from your manuscript), or elaborate on this item by providing additional information not in the ms, or briefly explain why the item is not applicable/relevant for your study

"The economic evaluation was performed alongside a single-blind parallel group two-arm RCT in which 288 participants aged between 18 and 65 years were eligible of whom 264 were randomized to either M-CT added to TAU or TAU alone (trialregister, identifier: NTR2503, approved by METIGG: an independent medical ethics committee)." + "The participants were randomized (allocation ratio 1:1)"

3b) Important changes to methods after trial commencement (such as eligibility criteria), with reasons

Does your paper address CONSORT subitem 3b? *

Copy and paste relevant sections from the manuscript (include quotes in quotation marks "like this" to indicate direct quotes from your manuscript), or elaborate on this item by providing additional information not in the ms, or briefly explain why the item is not applicable/relevant for your study

"Our initial criterion of having experienced two depressive episodes within 5 years was discarded, as individuals with multiple episodes over a longer period of time are also at risk [25]. We examined whether this affected our primary outcomes but this was not the case."

3b-i) Bug fixes, Downtimes, Content Changes

Bug fixes, Downtimes, Content Changes: ehealth systems are often dynamic systems. A description of changes to methods therefore also includes important changes made on the intervention or comparator during the trial (e.g., major bug fixes or changes in the functionality or content) (5-iii) and other “unexpected events” that may have influenced study design such as staff changes, system failures/downtimes, etc. [2].

	1	2	3	4	5
subitem not at					

You're editing your response. Sharing this URL allows others to also edit your response.

FILL IN A NEW RESPONSE

Does your paper address subitem 3b-i?

Copy and paste relevant sections from the manuscript (include quotes in quotation marks "like this" to indicate direct quotes from your manuscript), or elaborate on this item by providing additional information not in the ms, or briefly explain why the item is not applicable/relevant for your study

N/A

4a) Eligibility criteria for participants

Does your paper address CONSORT subitem 4a? *

Copy and paste relevant sections from the manuscript (include quotes in quotation marks "like this" to indicate direct quotes from your manuscript), or elaborate on this item by providing additional information not in the ms, or briefly explain why the item is not applicable/relevant for your study

"To be included, the following criteria had to be met: 1. A history of at least two Major Depressive Episodes (MDEs) according to the DSM-IV assessed with the Structured Clinical Interview for DSM-IV Disorders (SCID-I) [23] of which the latest MDE occurred within the last two years. 2. Currently remitted for at least 2 months according to the SCID-I and a score of ≤ 10 on the Hamilton Rating Scale for Depression (HRSD) [24]. Exclusion criteria were: current or past (hypo) mania, a bipolar or psychotic disorder, alcohol or drugs abuse, or a predominant anxiety disorder."

4a-i) Computer / Internet literacy

Computer / Internet literacy is often an implicit “de facto” eligibility criterion - this should be explicitly clarified.

	1	2	3	4	5	
subitem not at all important	○	○	○	○	○	essential

Does your paper address subitem 4a-i?

Copy and paste relevant sections from the manuscript (include quotes in quotation marks "like this" to indicate direct quotes from your manuscript), or elaborate on this item by providing additional information not in the ms, or briefly explain why the item is not applicable/relevant for your study

Computer literacy was not an inclusion criterion

You're editing your response. Sharing this URL allows others to also edit your response.

FILL IN A NEW RESPONSE

4a-ii) Open vs. closed, web-based vs. face-to-face assessments:

Open vs. closed, web-based vs. face-to-face assessments: Mention how participants were recruited (online vs. offline), e.g., from an open access website or from a clinic, and clarify if this was a purely web-based trial, or there were face-to-face components (as part of the intervention or for assessment), i.e., to what degree got the study team to know the participant. In online-only trials, clarify if participants were quasi-anonymous and whether having multiple identities was possible or whether technical or logistical measures (e.g., cookies, email confirmation, phone calls) were used to detect/prevent these.

	1	2	3	4	5	
subitem not at all important	○	○	○	○	○	essential

Does your paper address subitem 4a-ii? *

Copy and paste relevant sections from the manuscript (include quotes in quotation marks "like this" to indicate direct quotes from your manuscript), or elaborate on this item by providing additional information not in the ms, or briefly explain why the item is not applicable/relevant for your study

"The participants were recruited via media, general practitioners, and mental health care institutions"

4a-iii) Information giving during recruitment

Information given during recruitment. Specify how participants were briefed for recruitment and in the informed consent procedures (e.g., publish the informed consent documentation as appendix, see also item X26), as this information may have an effect on user self-selection, user expectation and may also bias results.

	1	2	3	4	5	
subitem not at all important	○	○	○	○	○	essential

Does your paper address subitem 4a-iii?

Copy and paste relevant sections from the manuscript (include quotes in quotation marks "like this" to indicate direct quotes from your manuscript), or elaborate on this item by providing additional information not in the ms, or briefly explain why the item is not applicable/relevant for your study

"The participants were recruited via media, general practitioners, and mental health care institutions and were included between mid-September 2010 and August 2013 after providing a written informed consent."

You're editing your response. Sharing this URL allows others to also edit your response.

FILL IN A NEW RESPONSE

Does your paper address CONSORT subitem 4b? *

Copy and paste relevant sections from the manuscript (include quotes in quotation marks "like this" to indicate direct quotes from your manuscript), or elaborate on this item by providing additional information not in the ms, or briefly explain why the item is not applicable/relevant for your study

"Cost data were collected with the Trimbos and iMTA questionnaire on Costs associated with Psychiatric illness (TiC-P) [33]. This questionnaire was administered online to all participants in 3-month intervals, starting at baseline."
"The health outcome measure of the cost-effectiveness analysis was the number of depression-free days based on DSM-IV criteria assessed with a telephone version of the SCID-I after 3, 12, and 24 months. Quality-Adjusted Life Years (QALYs) were used as health outcome measure of the cost-utility analysis using the area under the curve method. The QALY is a health measure that combines quality of life and the amount of time spent in a health condition, where one QALY is equal to 1 year lived in perfect health. The quality component of the QALY was derived from the EQ-5D-3L administered online in 3-month intervals starting from baseline [35] by using the algorithm of Dolan to obtain utilities for specific health states [36]."

4b-i) Report if outcomes were (self-)assessed through online questionnaires

Clearly report if outcomes were (self-)assessed through online questionnaires (as common in web-based trials) or otherwise.

	1	2	3	4	5	
subitem not at all important	○	○	○	○	○	essential

You're editing your response. Sharing this URL allows others to also edit your response.

FILL IN A NEW RESPONSE

Does your paper address subitem 4b-i? *

Copy and paste relevant sections from the manuscript (include quotes in quotation marks "like this" to indicate direct quotes from your manuscript), or elaborate on this item by providing additional information not in the ms, or briefly explain why the item is not applicable/relevant for your study

"Cost data were collected with the Trimbos and iMTA questionnaire on Costs associated with Psychiatric illness (TiC-P) [33]. This questionnaire was administered online to all participants in 3-month intervals, starting at baseline."
"The health outcome measure of the cost-effectiveness analysis was the number of depression-free days based on DSM-IV criteria assessed with a telephone version of the SCID-I after 3, 12, and 24 months. Quality-Adjusted Life Years (QALYs) were used as health outcome measure of the cost-utility analysis using the area under the curve method. The QALY is a health measure that combines quality of life and the amount of time spent in a health condition, where one QALY is equal to 1 year lived in perfect health. The quality component of the QALY was derived from the EQ-5D-3L administered online in 3-month intervals starting from baseline [35] by using the algorithm of Dolan to obtain utilities for specific health states [36]."

4b-ii) Report how institutional affiliations are displayed

Report how institutional affiliations are displayed to potential participants [on ehealth media], as affiliations with prestigious hospitals or universities may affect volunteer rates, use, and reactions with regards to an intervention.(Not a required item – describe only if this may bias results)

	1	2	3	4	5	
subitem not at all important	○	○	○	○	○	essential

Does your paper address subitem 4b-ii?

Copy and paste relevant sections from the manuscript (include quotes in quotation marks "like this" to indicate direct quotes from your manuscript), or elaborate on this item by providing additional information not in the ms, or briefly explain why the item is not applicable/relevant for your study

We used logos of the University in our information flyers

5) The interventions for each group with sufficient details to allow replication, including how and when they were actually

You're editing your response. Sharing this URL allows others to also edit your response.

FILL IN A NEW RESPONSE

5-i) Mention names, credential, affiliations of the developers, sponsors, and owners

Mention names, credential, affiliations of the developers, sponsors, and owners [6] (if authors/evaluators are owners or developer of the software, this needs to be declared in a “Conflict of interest” section or mentioned elsewhere in the manuscript).

	1	2	3	4	5	
subitem not at all important	○	○	○	○	○	essential

Does your paper address subitem 5-i?

Copy and paste relevant sections from the manuscript (include quotes in quotation marks "like this" to indicate direct quotes from your manuscript), or elaborate on this item by providing additional information not in the ms, or briefly explain why the item is not applicable/relevant for your study

"M-CT is based on Preventive Cognitive Therapy (PCT) [26], a face-to-face therapy that protects against relapse/recurrence in remitted individuals [27-29]. Bockting and Van Valen developed the content of M-CT [30] and it was built into the E-platform of the Trimbos Institute. Participants from previous relapse prevention studies and a patients’ association for depression (Depressievereniging) were involved in the development of the research question, outcome measures, the design, development, and implementation of M-CT."

"Conflicts of interest
Claudi L.H. Bockting and Evelien van Valen developed M-CT that was integrated in the platform of the Trimbos Institute in collaboration with Filip Smit. No other disclosures are reported."

5-ii) Describe the history/development process

Describe the history/development process of the application and previous formative evaluations (e.g., focus groups, usability testing), as these will have an impact on adoption/use rates and help with interpreting results.

	1	2	3	4	5	
subitem not at all important	○	○	○	○	○	essential

You're editing your response. Sharing this URL allows others to also edit your response.

FILL IN A NEW RESPONSE

Does your paper address subitem 5-ii?

Copy and paste relevant sections from the manuscript (include quotes in quotation marks "like this" to indicate direct quotes from your manuscript), or elaborate on this item by providing additional information not in the ms, or briefly explain why the item is not applicable/relevant for your study

"Participants from previous relapse prevention studies and a patients' association for depression (Depressievereniging) were involved in the development of the research question, outcome measures, the design, development, and implementation of M-CT."

5-iii) Revisions and updating

Revisions and updating. Clearly mention the date and/or version number of the application/intervention (and comparator, if applicable) evaluated, or describe whether the intervention underwent major changes during the evaluation process, or whether the development and/or content was “frozen” during the trial. Describe dynamic components such as news feeds or changing content which may have an impact on the replicability of the intervention (for unexpected events see item 3b).

	1	2	3	4	5	
subitem not at all important	○	○	○	○	○	essential

Does your paper address subitem 5-iii?

Copy and paste relevant sections from the manuscript (include quotes in quotation marks "like this" to indicate direct quotes from your manuscript), or elaborate on this item by providing additional information not in the ms, or briefly explain why the item is not applicable/relevant for your study

"The content of the program remained unaltered during the evaluation period"

5-iv) Quality assurance methods

Provide information on quality assurance methods to ensure accuracy and quality of information provided [1], if applicable.

	1	2	3	4	5	
subitem not at all important	○	○	○	○	○	essential

You're editing your response. Sharing this URL allows others to also edit your response.

FILL IN A NEW RESPONSE

Does your paper address subitem 5-iv?

Copy and paste relevant sections from the manuscript (include quotes in quotation marks "like this" to indicate direct quotes from your manuscript), or elaborate on this item by providing additional information not in the ms, or briefly explain why the item is not applicable/relevant for your study

"Participating therapists were supervised by an experienced clinical psychologist."

The study was coordinated and supervised by the research coordinator who took responsability for the quality assurance.

5-v) Ensure replicability by publishing the source code, and/or providing screenshots/screen-capture video, and/or providing flowcharts of the algorithms used

Ensure replicability by publishing the source code, and/or providing screenshots/screen-capture video, and/or providing flowcharts of the algorithms used. Replicability (i.e., other researchers should in principle be able to replicate the study) is a hallmark of scientific reporting.

	1	2	3	4	5	
subitem not at all important	○	○	○	○	○	essential

Does your paper address subitem 5-v?

Copy and paste relevant sections from the manuscript (include quotes in quotation marks "like this" to indicate direct quotes from your manuscript), or elaborate on this item by providing additional information not in the ms, or briefly explain why the item is not applicable/relevant for your study

Screenshots of the intervention have been published at Kok et al. (2014) Internet Interventions.

You're editing your response. Sharing this URL allows others to also edit your response.

FILL IN A NEW RESPONSE

5-vi) Digital preservation

Digital preservation: Provide the URL of the application, but as the intervention is likely to change or disappear over the course of the years; also make sure the intervention is archived (Internet Archive, webcitation.org, and/or publishing the source code or screenshots/videos alongside the article). As pages behind login screens cannot be archived, consider creating demo pages which are accessible without login.

	1	2	3	4	5	
subitem not at all important	○	○	○	○	○	essential

Does your paper address subitem 5-vi?

Copy and paste relevant sections from the manuscript (include quotes in quotation marks "like this" to indicate direct quotes from your manuscript), or elaborate on this item by providing additional information not in the ms, or briefly explain why the item is not applicable/relevant for your study

Screenshots of the intervention have been published at Kok et al. (2014) Internet Interventions.
The URL is: http://www.depressievrij.nl

5-vii) Access

Access: Describe how participants accessed the application, in what setting/context, if they had to pay (or were paid) or not, whether they had to be a member of specific group. If known, describe how participants obtained "access to the platform and Internet" [1]. To ensure access for editors/reviewers/readers, consider to provide a "backdoor" login account or demo mode for reviewers/readers to explore the application (also important for archiving purposes, see vi).

	1	2	3	4	5	
subitem not at all important	○	○	○	○	○	essential

Does your paper address subitem 5-vii? *

Copy and paste relevant sections from the manuscript (include quotes in quotation marks "like this" to indicate direct quotes from your manuscript), or elaborate on this item by providing additional information not in the ms, or briefly explain why the item is not applicable/relevant for your study

"The program was free of charge for the participants and an independent researcher provided participants with usernames and passwords to log in."

You're editing your response. Sharing this URL allows others to also edit your response.

FILL IN A NEW RESPONSE

5-viii) Mode of delivery, features/functionalities/components of the intervention and comparator, and the theoretical framework

Describe mode of delivery, features/functionalities/components of the intervention and comparator, and the theoretical framework [6] used to design them (instructional strategy [1], behaviour change techniques, persuasive features, etc., see e.g., [7, 8] for terminology). This includes an in-depth description of the content (including where it is coming from and who developed it) [1],” whether [and how] it is tailored to individual circumstances and allows users to track their progress and receive feedback” [6]. This also includes a description of communication delivery channels and – if computer-mediated communication is a component – whether communication was synchronous or asynchronous [6]. It also includes information on presentation strategies [1], including page design principles, average amount of text on pages, presence of hyperlinks to other resources, etc. [1].

	1	2	3	4	5	
subitem not at all important	○	○	○	○	○	essential

You're editing your response. Sharing this URL allows others to also edit your response.

FILL IN A NEW RESPONSE

Does your paper address subitem 5-viii? *

Copy and paste relevant sections from the manuscript (include quotes in quotation marks "like this" to indicate direct quotes from your manuscript), or elaborate on this item by providing additional information not in the ms, or briefly explain why the item is not applicable/relevant for your study

"M-CT is based on Preventive Cognitive Therapy (PCT) [26], a face-to-face therapy that protects against relapse/recurrence in remitted individuals [27-29]. Bockting and Van Valen developed the content of M-CT [30] and it was built into the E-platform of the Trimbos Institute. Participants from previous relapse prevention studies and a patients' association for depression (Depressievereniging) were involved in the development of the research question, outcome measures, the design, development, and implementation of M-CT. The content of the program remained unaltered during the evaluation period and logfile analysis was used to monitor the use of the intervention. The program was free of charge for the participants and an independent researcher provided participants with usernames and passwords to log in. M-CT comprised eight online modules with minimal therapist support and continued mobile mood monitoring using text messages. The participants were advised to work on one lesson each week and were offered a minimum of two and a maximum of four telephone contacts with a therapist (maximum duration: 30 minutes per contact). Two therapist contacts were pre-booked and two optional contacts could be booked by the participants additionally. Participating therapists were supervised by an experienced clinical psychologist. The primary task of the therapists was to work through the M-CT program. The participants received a reminder per e-mail or text message if they did not log into the website for 6 weeks. Feedback from the participants on the intervention was obtained by giving them the opportunity to evaluate each specific lesson. Participants randomized to M-CT and TAU continued to receive usual care, which could include for example ADs, counseling, or no care."

5-ix) Describe use parameters

Describe use parameters (e.g., intended "doses" and optimal timing for use). Clarify what instructions or recommendations were given to the user, e.g., regarding timing, frequency, heaviness of use, if any, or was the intervention used ad libitum.

	1	2	3	4	5	
subitem not at	○	○	○	○	○	[illegible]

You're editing your response. Sharing this URL allows others to also edit your response.

FILL IN A NEW RESPONSE

Does your paper address subitem 5-ix?

Copy and paste relevant sections from the manuscript (include quotes in quotation marks "like this" to indicate direct quotes from your manuscript), or elaborate on this item by providing additional information not in the ms, or briefly explain why the item is not applicable/relevant for your study

"The participants were advised to work on one lesson each week and were offered a minimum of two and a maximum of four telephone contacts with a therapist (maximum duration: 30 minutes per contact). Two therapist contacts were pre-booked and two optional contacts could be booked by the participants additionally."

5-x) Clarify the level of human involvement

Clarify the level of human involvement (care providers or health professionals, also technical assistance) in the e-intervention or as co-intervention (detail number and expertise of professionals involved, if any, as well as “type of assistance offered, the timing and frequency of the support, how it is initiated, and the medium by which the assistance is delivered”. It may be necessary to distinguish between the level of human involvement required for the trial, and the level of human involvement required for a routine application outside of a RCT setting (discuss under item 21 – generalizability).

	1	2	3	4	5	
subitem not at all important	○	○	○	○	○	essential

Does your paper address subitem 5-x?

Copy and paste relevant sections from the manuscript (include quotes in quotation marks "like this" to indicate direct quotes from your manuscript), or elaborate on this item by providing additional information not in the ms, or briefly explain why the item is not applicable/relevant for your study

"The participants were advised to work on one lesson each week and were offered a minimum of two and a maximum of four telephone contacts with a therapist (maximum duration: 30 minutes per contact). Two therapist contacts were pre-booked and two optional contacts could be booked by the participants additionally. Participating therapists were supervised by an experienced clinical psychologist. The primary task of the therapists was to work through the M-CT program."

You're editing your response. Sharing this URL allows others to also edit your response.

FILL IN A NEW RESPONSE

5-xi) Report any prompts/reminders used

Report any prompts/reminders used: Clarify if there were prompts (letters, emails, phone calls, SMS) to use the application, what triggered them, frequency etc. It may be necessary to distinguish between the level of prompts/reminders required for the trial, and the level of prompts/reminders for a routine application outside of a RCT setting (discuss under item 21 – generalizability).

	1	2	3	4	5	
subitem not at all important	○	○	○	○	○	essential

Does your paper address subitem 5-xi? *

Copy and paste relevant sections from the manuscript (include quotes in quotation marks "like this" to indicate direct quotes from your manuscript), or elaborate on this item by providing additional information not in the ms, or briefly explain why the item is not applicable/relevant for your study

"The participants received a reminder per e-mail or text message if they did not log into the website for 6 weeks."

5-xii) Describe any co-interventions (incl. training/support)

Describe any co-interventions (incl. training/support): Clearly state any interventions that are provided in addition to the targeted eHealth intervention, as ehealth intervention may not be designed as stand-alone intervention. This includes training sessions and support [1]. It may be necessary to distinguish between the level of training required for the trial, and the level of training for a routine application outside of a RCT setting (discuss under item 21 – generalizability.

	1	2	3	4	5	
subitem not at all important	○	○	○	○	○	essential

Does your paper address subitem 5-xii? *

Copy and paste relevant sections from the manuscript (include quotes in quotation marks "like this" to indicate direct quotes from your manuscript), or elaborate on this item by providing additional information not in the ms, or briefly explain why the item is not applicable/relevant for your study

"Two therapist contacts were pre-booked and two optional contacts could be booked by the participants additionally. Participating therapists were supervised by an experienced clinical psychologist. The primary task of the therapists was to work through the M-CT program."

You're editing your response. Sharing this URL allows others to also edit your response.

FILL IN A NEW RESPONSE

6a) Completely defined pre-specified primary and secondary outcome measures, including how and when they were assessed

Does your paper address CONSORT subitem 6a? *

Copy and paste relevant sections from the manuscript (include quotes in quotation marks "like this" to indicate direct quotes from your manuscript), or elaborate on this item by providing additional information not in the ms, or briefly explain why the item is not applicable/relevant for your study

"Cost data were collected with the Trimbos and iMTA questionnaire on Costs associated with Psychiatric illness (TiC-P) [33]. This questionnaire was administered online to all participants in 3-month intervals, starting at baseline."

"The health outcome measure of the cost-effectiveness analysis was the number of depression-free days based on DSM-IV criteria assessed with a telephone version of the SCID-I after 3, 12, and 24 months."

"The quality component of the QALY was derived from the EQ-5D-3L administered online in 3-month intervals starting from baseline [35] by using the algorithm of Dolan to obtain utilities for specific health states [36]. The EQ-5D-3L is a commonly applied self-administered instrument that measures the generic health status and consists of five questions covering the following five dimensions: mobility, self-care, usual activities, pain/discomfort, and anxiety/depression."

6a-i) Online questionnaires: describe if they were validated for online use and apply CHERRIES items to describe how the questionnaires were designed/deployed

If outcomes were obtained through online questionnaires, describe if they were validated for online use and apply CHERRIES items to describe how the questionnaires were designed/deployed [9].

	1	2	3	4	5	
subitem not at all important	○	○	○	○	○	essential

You're editing your response. Sharing this URL allows others to also edit your response.

FILL IN A NEW RESPONSE

Does your paper address subitem 6a-i?

Copy and paste relevant sections from manuscript text

The EQ-5D and TiC-P have been validated:
e.g., BMC Health Serv Res. 2013;13(1):217, Eur J Health Econ. 2017;18(4):519-531

6a-ii) Describe whether and how “use” (including intensity of use/dosage) was defined/measured/monitored

Describe whether and how “use” (including intensity of use/dosage) was defined/measured/monitored (logins, logfile analysis, etc.). Use/adoption metrics are important process outcomes that should be reported in any ehealth trial.

	1	2	3	4	5	
subitem not at all important	○	○	○	○	○	essential

Does your paper address subitem 6a-ii?

Copy and paste relevant sections from manuscript text

"The content of the program remained unaltered during the evaluation period and logfile analysis was used to monitor the use of the intervention. "

6a-iii) Describe whether, how, and when qualitative feedback from participants was obtained

Describe whether, how, and when qualitative feedback from participants was obtained (e.g., through emails, feedback forms, interviews, focus groups).

	1	2	3	4	5	
subitem not at all important	○	○	○	○	○	essential

Does your paper address subitem 6a-iii?

Copy and paste relevant sections from manuscript text

"Feedback from the participants on the intervention was obtained by giving them the opportunity to evaluate each specific lesson."

You're editing your response. Sharing this URL allows others to also edit your response.

FILL IN A NEW RESPONSE

6b) Any changes to trial outcomes after the trial commenced, with reasons

Does your paper address CONSORT subitem 6b? *

Copy and paste relevant sections from the manuscript (include quotes in quotation marks "like this" to indicate direct quotes from your manuscript), or elaborate on this item by providing additional information not in the ms, or briefly explain why the item is not applicable/relevant for your study

"Our initial criterion of having experienced two depressive episodes within 5 years was discarded, as individuals with multiple episodes over a longer period of time are also at risk [25]. We examined whether this affected our primary outcomes but this was not the case."

7a) How sample size was determined

NPT: When applicable, details of whether and how the clustering by care provides or centers was addressed

7a-i) Describe whether and how expected attrition was taken into account when calculating the sample size

Describe whether and how expected attrition was taken into account when calculating the sample size.

	1	2	3	4	5	
subitem not at all important	○	○	○	○	○	essential

Does your paper address subitem 7a-i?

Copy and paste relevant sections from manuscript title (include quotes in quotation marks "like this" to indicate direct quotes from your manuscript), or elaborate on this item by providing additional information not in the ms, or briefly explain why the item is not applicable/relevant for your study

"The power calculation of the primary outcome is described elsewhere [20,22]. Since the study was only powered to detect differences in health outcomes and not in costs, as in most economic evaluations, we used probabilistic and medical decision-making techniques to draw inferences about the cost-effectiveness."

You're editing your response. Sharing this URL allows others to also edit your response.

FILL IN A NEW RESPONSE

7b) When applicable, explanation of any interim analyses and stopping guidelines

Does your paper address CONSORT subitem 7b? *

Copy and paste relevant sections from the manuscript (include quotes in quotation marks "like this" to indicate direct quotes from your manuscript), or elaborate on this item by providing additional information not in the ms, or briefly explain why the item is not applicable/relevant for your study

N/A

8a) Method used to generate the random allocation sequence

NPT: When applicable, how care providers were allocated to each trial group

Does your paper address CONSORT subitem 8a? *

Copy and paste relevant sections from the manuscript (include quotes in quotation marks "like this" to indicate direct quotes from your manuscript), or elaborate on this item by providing additional information not in the ms, or briefly explain why the item is not applicable/relevant for your study

"Randomization was planned to be stratified by type of aftercare and number of MDEs, but eventually simple randomization was carried out due to a programming error. The participants were randomized (allocation ratio 1:1) by an independent researcher not otherwise involved in the study who was masked for clinical characteristics and who used computer-generated numbers in STATA. An independent researcher not involved in the follow-up interviews assigned the participants to the treatment conditions. The participants were not blinded to treatment allocation due to the nature of the intervention. The interviewers were unaware of the participants' treatment allocation and the participants were instructed not to inform the interviewer of their treatment allocation. The assessor was replaced by another independent assessor in case the randomization was broken."

8b) Type of randomisation; details of any restriction (such as blocking and block size)

You're editing your response. Sharing this URL allows others to also edit your response.

FILL IN A NEW RESPONSE

Does your paper address CONSORT subitem 8b? *

Copy and paste relevant sections from the manuscript (include quotes in quotation marks "like this" to indicate direct quotes from your manuscript), or elaborate on this item by providing additional information not in the ms, or briefly explain why the item is not applicable/relevant for your study

"Randomization was planned to be stratified by type of aftercare and number of MDEs, but eventually simple randomization was carried out due to a programming error."

9) Mechanism used to implement the random allocation sequence (such as sequentially numbered containers), describing any steps taken to conceal the sequence until interventions were assigned

Does your paper address CONSORT subitem 9? *

Copy and paste relevant sections from the manuscript (include quotes in quotation marks "like this" to indicate direct quotes from your manuscript), or elaborate on this item by providing additional information not in the ms, or briefly explain why the item is not applicable/relevant for your study

"The participants were randomized (allocation ratio 1:1) by an independent researcher not otherwise involved in the study who was masked for clinical characteristics and who used computer-generated numbers in STATA."

10) Who generated the random allocation sequence, who enrolled participants, and who assigned participants to interventions

You're editing your response. Sharing this URL allows others to also edit your response.

FILL IN A NEW RESPONSE

Does your paper address CONSORT subitem 10? *

Copy and paste relevant sections from the manuscript (include quotes in quotation marks "like this" to indicate direct quotes from your manuscript), or elaborate on this item by providing additional information not in the ms, or briefly explain why the item is not applicable/relevant for your study

"Independent psychologists or research assistants interviewed the potential participants for inclusion and exclusion criteria."

"The participants were randomized (allocation ratio 1:1) by an independent researcher not otherwise involved in the study who was masked for clinical characteristics and who used computer-generated numbers in STATA."

"An independent researcher not involved in the follow-up interviews assigned the participants to the treatment conditions."

11a) If done, who was blinded after assignment to interventions (for example, participants, care providers, those assessing outcomes) and how

NPT: Whether or not administering co-interventions were blinded to group assignment

11a-i) Specify who was blinded, and who wasn’t

Specify who was blinded, and who wasn’t. Usually, in web-based trials it is not possible to blind the participants [1, 3] (this should be clearly acknowledged), but it may be possible to blind outcome assessors, those doing data analysis or those administering co-interventions (if any).

	1	2	3	4	5	
subitem not at all important	○	○	○	○	○	essential

You're editing your response. Sharing this URL allows others to also edit your response.

FILL IN A NEW RESPONSE

Does your paper address subitem 11a-i? *

Copy and paste relevant sections from the manuscript (include quotes in quotation marks "like this" to indicate direct quotes from your manuscript), or elaborate on this item by providing additional information not in the ms, or briefly explain why the item is not applicable/relevant for your study

"An independent researcher not involved in the follow-up interviews assigned the participants to the treatment conditions. The participants were not blinded to treatment allocation due to the nature of the intervention. The interviewers were unaware of the participants' treatment allocation and the participants were instructed not to inform the interviewer of their treatment allocation. The assessor was replaced by another independent assessor in case the randomization was broken."

11a-ii) Discuss e.g., whether participants knew which intervention was the "intervention of interest" and which one was the "comparator"

Informed consent procedures (4a-ii) can create biases and certain expectations - discuss e.g., whether participants knew which intervention was the "intervention of interest" and which one was the "comparator".

	1	2	3	4	5	
subitem not at all important	○	○	○	○	○	essential

Does your paper address subitem 11a-ii?

Copy and paste relevant sections from the manuscript (include quotes in quotation marks "like this" to indicate direct quotes from your manuscript), or elaborate on this item by providing additional information not in the ms, or briefly explain why the item is not applicable/relevant for your study

"The participants were not blinded to treatment allocation due to the nature of the intervention."

11b) If relevant, description of the similarity of interventions

(this item is usually not relevant for ehealth trials as it refers to similarity of a placebo or sham intervention to a active medication/intervention)

You're editing your response. Sharing this URL allows others to also edit your response.

FILL IN A NEW RESPONSE

Does your paper address CONSORT subitem 11b? *

Copy and paste relevant sections from the manuscript (include quotes in quotation marks "like this" to indicate direct quotes from your manuscript), or elaborate on this item by providing additional information not in the ms, or briefly explain why the item is not applicable/relevant for your study

N/A

12a) Statistical methods used to compare groups for primary and secondary outcomes

NPT: When applicable, details of whether and how the clustering by care providers or centers was addressed

You're editing your response. Sharing this URL allows others to also edit your response.

FILL IN A NEW RESPONSE

Does your paper address CONSORT subitem 12a? *

Copy and paste relevant sections from the manuscript (include quotes in quotation marks "like this" to indicate direct quotes from your manuscript), or elaborate on this item by providing additional information not in the ms, or briefly explain why the item is not applicable/relevant for your study

"Quality-Adjusted Life Years (QALYs) were used as health outcome measure of the cost-utility analysis using the area under the curve method. The QALY is a health measure that combines quality of life and the amount of time spent in a health condition, where one QALY is equal to 1 year lived in perfect health. The quality component of the QALY was derived from the EQ-5D-3L administered online in 3-month intervals starting from baseline [35] by using the algorithm of Dolan to obtain utilities for specific health states [36]."

"The economic evaluation was conducted and reported according to the Consolidated Health Economic Evaluation Reporting Standards statement (CHEERS) (Multimedia Appendix 2)."

"Costs and outcomes were used to calculate the Incremental Cost-Effectiveness Ratio (ICER) of M-CT relative to TAU alone [40]. The formula used for calculating the ICER is:
where, C M-CT and C TAU are the mean costs, and QALY M-CT and QALY TAU are the mean QALYs in M-CT and TAU, respectively. The ICER is interpreted as the additional costs per QALY gained when M-CT is offered rather than TAU. The bootstrap method [41] was applied to account for the uncertainty in the economic evaluation by repeated random sampling with replacement from the original dataset. Seemingly Unrelated Regression Equations (SURE) were bootstrapped (5000 times) to allow for correlated residuals of the cost- and utility equations and to adjust for baseline differences in one of the sensitivity analyses. In each bootstrap step, the mean cost differences and the mean outcome differences were computed and these were plotted in the cost-effectiveness plane [42]. Finally, Cost-Effectiveness Acceptability Curves (CEACs) [43] were graphed, taking into account the relative placement of the bootstrap replications. CEACs inform decision-makers on the likelihood that an intervention is deemed to be cost-effective given a range of willingness-to-pay ceilings for gaining and additional unit of health (i.e., gaining one QALY and gaining one depression-free day). The analyses were conducted using Stata."

You're editing your response. Sharing this URL allows others to also edit your response.

FILL IN A NEW RESPONSE

12a-i) Imputation techniques to deal with attrition / missing values

Imputation techniques to deal with attrition / missing values: Not all participants will use the intervention/comparator as intended and attrition is typically high in ehealth trials. Specify how participants who did not use the application or dropped out from the trial were treated in the statistical analysis (a complete case analysis is strongly discouraged, and simple imputation techniques such as LOCF may also be problematic [4]).

	1	2	3	4	5	
subitem not at all important	○	○	○	○	○	essential

Does your paper address subitem 12a-i? *

Copy and paste relevant sections from the manuscript (include quotes in quotation marks "like this" to indicate direct quotes from your manuscript), or elaborate on this item by providing additional information not in the ms, or briefly explain why the item is not applicable/relevant for your study

"In our main analysis, we used multiple imputations by chained equations with predictive mean matching to account for missing data. The use of this technique may avoid bias associated with complete case analyses and makes optimal use of available data. Baseline variables predictive of clinical and cost outcomes and of a variable being missing were incorporated in the imputation model as recommended by White et al. [37] to enhance the precision of the model and to correct for possible bias. To account for participants with extremely high costs resulting in unstable imputation estimates, winsorizing was used for the main analyses. Using winsorizing, extreme values are instead replaced by certain percentiles, in this case the 97.5th percentile as opposed to trimming in which the extreme values are merely deleted [38,39]."

12b) Methods for additional analyses, such as subgroup analyses and adjusted analyses

You're editing your response. Sharing this URL allows others to also edit your response.

FILL IN A NEW RESPONSE

Does your paper address CONSORT subitem 12b? *

Copy and paste relevant sections from the manuscript (include quotes in quotation marks "like this" to indicate direct quotes from your manuscript), or elaborate on this item by providing additional information not in the ms, or briefly explain why the item is not applicable/relevant for your study

"Due to the amount of missing data, we used multiple imputations in the main analysis to handle missing data. To ascertain the robustness of our findings, we performed several sensitivity analyses, each handling missing data in a different way. It should be noted that in the main analysis, 29 participants were not included since they dropped-out immediately after randomization and therefore no follow-up information was available. Nevertheless, we performed an additional sensitivity analysis in which all participants were included for a full-fledged intention-to-treat analysis. The main analysis was repeated again but now restricted to individuals for whom at least 50% of the cost data were available. A final analysis to evaluate the impact of drop-out was based on complete cases. At baseline, we observed a slight imbalance between both conditions with respect to gender, severity of the last MDE, and baseline costs. Studies suggest that gender is not associated with relapse or recurrence but that severity of the last MDE might be [25]. Therefore, we repeated the main analysis but now adjusting for the small baseline imbalances in severity of the last MDE and baseline costs."

X26) REB/IRB Approval and Ethical Considerations [recommended as subheading under "Methods"] (not a CONSORT item)

X26-i) Comment on ethics committee approval

	1	2	3	4	5	
subitem not at all important	○	○	○	○	○	essential

Does your paper address subitem X26-i?

Copy and paste relevant sections from the manuscript (include quotes in quotation marks "like this" to indicate direct quotes from your manuscript), or elaborate on this item by providing additional information not in the ms, or briefly explain why the item is not applicable/relevant for your study

You're editing your response. Sharing this URL allows others to also edit your response.

FILL IN A NEW RESPONSE

x26-ii) Outline informed consent procedures

Outline informed consent procedures e.g., if consent was obtained offline or online (how? Checkbox, etc.?), and what information was provided (see 4a-ii). See [6] for some items to be included in informed consent documents.

	1	2	3	4	5	
subitem not at all important	○	○	○	○	○	essential

Does your paper address subitem X26-ii?

Copy and paste relevant sections from the manuscript (include quotes in quotation marks "like this" to indicate direct quotes from your manuscript), or elaborate on this item by providing additional information not in the ms, or briefly explain why the item is not applicable/relevant for your study

"The participants were recruited via media, general practitioners, and mental health care institutions and were included between mid-September 2010 and August 2013 after providing a written informed consent."

X26-iii) Safety and security procedures

Safety and security procedures, incl. privacy considerations, and any steps taken to reduce the likelihood or detection of harm (e.g., education and training, availability of a hotline)

	1	2	3	4	5	
subitem not at all important	○	○	○	○	○	essential

Does your paper address subitem X26-iii?

Copy and paste relevant sections from the manuscript (include quotes in quotation marks "like this" to indicate direct quotes from your manuscript), or elaborate on this item by providing additional information not in the ms, or briefly explain why the item is not applicable/relevant for your study

Adverse events were monitored using the SCID and the online IDS-SR.

RESULTS

You're editing your response. Sharing this URL allows others to also edit your response.

FILL IN A NEW RESPONSE

13a) For each group, the numbers of participants who were randomly assigned, received intended treatment, and were analysed for the primary outcome

NPT: The number of care providers or centers performing the intervention in each group and the number of patients treated by each care provider in each center

Does your paper address CONSORT subitem 13a? *

Copy and paste relevant sections from the manuscript (include quotes in quotation marks "like this" to indicate direct quotes from your manuscript), or elaborate on this item by providing additional information not in the ms, or briefly explain why the item is not applicable/relevant for your study

"In total, 288 participants were eligible of whom 264 were randomized to either M-CT added to TAU (n = 132) or TAU alone (n = 132). In total, 29 participants dropped-out immediately after randomization and 24 were lost to follow-up during the study."

"It should be noted that in the main analysis, 29 participants were not included since they dropped-out immediately after randomization and therefore no follow-up information was available."

13b) For each group, losses and exclusions after randomisation, together with reasons

You're editing your response. Sharing this URL allows others to also edit your response.

FILL IN A NEW RESPONSE

Does your paper address CONSORT subitem 13b? (NOTE: Preferably, this is shown in a CONSORT flow diagram) *

Copy and paste relevant sections from the manuscript (include quotes in quotation marks "like this" to indicate direct quotes from your manuscript), or elaborate on this item by providing additional information not in the ms, or briefly explain why the item is not applicable/relevant for your study

"It should be noted that in the main analysis, 29 participants were not included since they dropped-out immediately after randomization and therefore no follow-up information was available."

"The participant flow during the study is displayed in Multimedia Appendix 3. In total, 288 participants were eligible of whom 264 were randomized to either M-CT added to TAU (n = 132) or TAU alone (n = 132). In total, 29 participants dropped-out immediately after randomization and 24 were lost to follow-up during the study. Overall, the baseline clinical and demographic characteristics of all participants (Table 1) and participants with any follow-up data were similar and equally distributed over the treatment conditions, suggesting no systematic bias owing to drop-out of the 29 individuals with no follow-up data. Complete cost and effect data (available for all measurements) were available for 45 participants (17%). At least one measurement of cost data after baseline was available for 195 (75%) of the participants. For 129 participants (49%), at least half of the cost measurements were available."

13b-i) Attrition diagram

Strongly recommended: An attrition diagram (e.g., proportion of participants still logging in or using the intervention/comparator in each group plotted over time, similar to a survival curve) or other figures or tables demonstrating usage/dose/engagement.

	1	2	3	4	5	
subitem not at all important	○	○	○	○	○	essential

You're editing your response. Sharing this URL allows others to also edit your response.

FILL IN A NEW RESPONSE

Does your paper address subitem 13b-i?

Copy and paste relevant sections from the manuscript or cite the figure number if applicable (include quotes in quotation marks "like this" to indicate direct quotes from your manuscript), or elaborate on this item by providing additional information not in the ms, or briefly explain why the item is not applicable/relevant for your study

"The participant flow during the study is displayed in Multimedia Appendix 3."

"As reported elsewhere [20], treatment adherence in the current study was comparable to other studies (in total, 90 out of 132 (68.2%) finished at least five lessons) [15] and therefore we believe this did not explain the results."

14a) Dates defining the periods of recruitment and follow-up

Does your paper address CONSORT subitem 14a? *

Copy and paste relevant sections from the manuscript (include quotes in quotation marks "like this" to indicate direct quotes from your manuscript), or elaborate on this item by providing additional information not in the ms, or briefly explain why the item is not applicable/relevant for your study

"The participants were recruited via media, general practitioners, and mental health care institutions and were included between mid-September 2010 and August 2013 after providing a written informed consent."

14a-i) Indicate if critical “secular events” fell into the study period

Indicate if critical “secular events” fell into the study period, e.g., significant changes in Internet resources available or “changes in computer hardware or Internet delivery resources”

	1	2	3	4	5	
subitem not at all important	○	○	○	○	○	essential

Does your paper address subitem 14a-i?

Copy and paste relevant sections from the manuscript (include quotes in quotation marks "like this" to indicate direct quotes from your manuscript), or elaborate on this item by providing additional information not in the ms, or briefly explain why the item is not applicable/relevant for your study

N/A

You're editing your response. Sharing this URL allows others to also edit your response.

FILL IN A NEW RESPONSE

Does your paper address CONSORT subitem 14b? *

Copy and paste relevant sections from the manuscript (include quotes in quotation marks "like this" to indicate direct quotes from your manuscript), or elaborate on this item by providing additional information not in the ms, or briefly explain why the item is not applicable/relevant for your study

N/A

15) A table showing baseline demographic and clinical characteristics for each group

NPT: When applicable, a description of care providers (case volume, qualification, expertise, etc.) and centers (volume) in each group

Does your paper address CONSORT subitem 15? *

Copy and paste relevant sections from the manuscript (include quotes in quotation marks "like this" to indicate direct quotes from your manuscript), or elaborate on this item by providing additional information not in the ms, or briefly explain why the item is not applicable/relevant for your study

Table 1

15-i) Report demographics associated with digital divide issues

In ehealth trials it is particularly important to report demographics associated with digital divide issues, such as age, education, gender, social-economic status, computer/Internet/ehealth literacy of the participants, if known.

	1	2	3	4	5	
subitem not at all important	○	○	○	○	○	essential

Does your paper address subitem 15-i? *

Copy and paste relevant sections from the manuscript (include quotes in quotation marks "like this" to indicate direct quotes from your manuscript), or elaborate on this item by providing additional information not in the ms, or briefly explain why the item is not applicable/relevant for your study

Table 1

16) For each group, number of participants (denominator) [illegible]

You're editing your response. Sharing this URL allows others to also edit your response.

FILL IN A NEW RESPONSE

16-i) Report multiple “denominators” and provide definitions

Report multiple “denominators” and provide definitions: Report N’s (and effect sizes) “across a range of study participation [and use] thresholds” [1], e.g., N exposed, N consented, N used more than x times, N used more than y weeks, N participants “used” the intervention/comparator at specific pre-defined time points of interest (in absolute and relative numbers per group). Always clearly define “use” of the intervention.

	1	2	3	4	5	
subitem not at all important	○	○	○	○	○	essential

Does your paper address subitem 16-i? *

Copy and paste relevant sections from the manuscript (include quotes in quotation marks "like this" to indicate direct quotes from your manuscript), or elaborate on this item by providing additional information not in the ms, or briefly explain why the item is not applicable/relevant for your study

"The intention-to-treat principle was used, in which all participants were analysed according to their randomized condition, irrespective of their actual treatment."

16-ii) Primary analysis should be intent-to-treat

Primary analysis should be intent-to-treat, secondary analyses could include comparing only “users”, with the appropriate caveats that this is no longer a randomized sample (see 18-i).

	1	2	3	4	5	
subitem not at all important	○	○	○	○	○	essential

Does your paper address subitem 16-ii?

Copy and paste relevant sections from the manuscript (include quotes in quotation marks "like this" to indicate direct quotes from your manuscript), or elaborate on this item by providing additional information not in the ms, or briefly explain why the item is not applicable/relevant for your study

"The intention-to-treat principle was used, in which all participants were analysed according to their randomized condition, irrespective of their actual treatment."

17a) For each primary and secondary outcome, results for each

You're editing your response. Sharing this URL allows others to also edit your response.

FILL IN A NEW RESPONSE

Does your paper address CONSORT subitem 17a? *

Copy and paste relevant sections from the manuscript (include quotes in quotation marks "like this" to indicate direct quotes from your manuscript), or elaborate on this item by providing additional information not in the ms, or briefly explain why the item is not applicable/relevant for your study

It is an economic evaluation, we present all the relevant data in Table 2 and Figure 1 and 2 and describe in the text:

"According to the main analysis, M-CT resulted in slightly better health outcomes (an extra 5.6 gain in depression-free days, 95% CI: 5.3 – 6.0, and a 0.004 QALY gain, 95% CI: 0.004 – 0.005), but these health gains were achieved at higher costs (€1,008, 95% CI: €983 – €1,034). For both health outcomes, most of the bootstrapped ICERs were located in the North-East quadrant (55.5% for depression-free days and 46.5% for QALYs), indicating that the probability that M-CT is deemed cost-effective depends on the willingness-to-pay for an additional health gain (see Figure 1 and 2). When the willingness-to-pay per additional depression-free day is zero, M-CT has approximately a 40% probability to be cost-effective. When the willingness-to-pay per additional gain in depression-free days increases, the probability that M-CT is cost-effective also increases but does not rise above 65%. For QALYs, increased willingness-to-pay only leads to slight increases in the probability that M-CT will be considered cost-effective and the probability that M-CT is cost-effective does not rise above 40%."

17a-i) Presentation of process outcomes such as metrics of use and intensity of use

In addition to primary/secondary (clinical) outcomes, the presentation of process outcomes such as metrics of use and intensity of use (dose, exposure) and their operational definitions is critical. This does not only refer to metrics of attrition (13-b) (often a binary variable), but also to more continuous exposure metrics such as “average session length”. These must be accompanied by a technical description how a metric like a “session” is defined (e.g., timeout after idle time) [1] (report under item 6a).

	1	2	3	4	5	
subitem not at all important	○	○	○	○	○	essential

You're editing your response. Sharing this URL allows others to also edit your response.

FILL IN A NEW RESPONSE

Does your paper address subitem 17a-i?

Copy and paste relevant sections from the manuscript (include quotes in quotation marks "like this" to indicate direct quotes from your manuscript), or elaborate on this item by providing additional information not in the ms, or briefly explain why the item is not applicable/relevant for your study

"As reported elsewhere [20], treatment adherence in the current study was comparable to other studies (in total, 90 out of 132 (68.2%) finished at least five lessons) [15] and therefore we believe this did not explain the results."

17b) For binary outcomes, presentation of both absolute and relative effect sizes is recommended

Does your paper address CONSORT subitem 17b? *

Copy and paste relevant sections from the manuscript (include quotes in quotation marks "like this" to indicate direct quotes from your manuscript), or elaborate on this item by providing additional information not in the ms, or briefly explain why the item is not applicable/relevant for your study

N/A: it is an economic evaluation

18) Results of any other analyses performed, including subgroup analyses and adjusted analyses, distinguishing pre-specified from exploratory

Does your paper address CONSORT subitem 18? *

Copy and paste relevant sections from the manuscript (include quotes in quotation marks "like this" to indicate direct quotes from your manuscript), or elaborate on this item by providing additional information not in the ms, or briefly explain why the item is not applicable/relevant for your study

"Table 2 displays the main analysis and sensitivity analyses. The sensitivity analysis including all randomized participants (n = 264) overall yielded similar results. When taking into account participants for whom at least 50% of the data were available, TAU dominated M-CT in terms of depression-free days and results were roughly similar to the main analysis for QALYs. In the complete case analysis, TAU dominated M-CT. Adjustments for imbalanced baseline variables yielded similar results."

You're editing your response. Sharing this URL allows others to also edit your response.

FILL IN A NEW RESPONSE

18-i) Subgroup analysis of comparing only users

A subgroup analysis of comparing only users is not uncommon in ehealth trials, but if done, it must be stressed that this is a self-selected sample and no longer an unbiased sample from a randomized trial (see 16-iii).

	1	2	3	4	5	
subitem not at all important	○	○	○	○	○	essential

Does your paper address subitem 18-i?

Copy and paste relevant sections from the manuscript (include quotes in quotation marks "like this" to indicate direct quotes from your manuscript), or elaborate on this item by providing additional information not in the ms, or briefly explain why the item is not applicable/relevant for your study

N/A: this is an economic evaluation

19) All important harms or unintended effects in each group

(for specific guidance see CONSORT for harms)

Does your paper address CONSORT subitem 19? *

Copy and paste relevant sections from the manuscript (include quotes in quotation marks "like this" to indicate direct quotes from your manuscript), or elaborate on this item by providing additional information not in the ms, or briefly explain why the item is not applicable/relevant for your study

N/A

19-i) Include privacy breaches, technical problems

Include privacy breaches, technical problems. This does not only include physical "harm" to participants, but also incidents such as perceived or real privacy breaches [1], technical problems, and other unexpected/unintended incidents. "Unintended effects" also includes unintended positive effects [2].

	1	2	3	4	5	
subitem not at all important	○	○	○	○	○	essential

You're editing your response. Sharing this URL allows others to also edit your response.

FILL IN A NEW RESPONSE

Does your paper address subitem 19-i?

Copy and paste relevant sections from the manuscript (include quotes in quotation marks "like this" to indicate direct quotes from your manuscript), or elaborate on this item by providing additional information not in the ms, or briefly explain why the item is not applicable/relevant for your study

N/A

19-ii) Include qualitative feedback from participants or observations from staff/researchers

Include qualitative feedback from participants or observations from staff/researchers, if available, on strengths and shortcomings of the application, especially if they point to unintended/unexpected effects or uses. This includes (if available) reasons for why people did or did not use the application as intended by the developers.

	1	2	3	4	5	
subitem not at all important	○	○	○	○	○	essential

Does your paper address subitem 19-ii?

Copy and paste relevant sections from the manuscript (include quotes in quotation marks "like this" to indicate direct quotes from your manuscript), or elaborate on this item by providing additional information not in the ms, or briefly explain why the item is not applicable/relevant for your study

N/A: this is an economic evaluation

DISCUSSION

22) Interpretation consistent with results, balancing benefits and harms, and considering other relevant evidence

NPT: In addition, take into account the choice of the comparator, lack of or partial blinding, and unequal expertise of care providers or centers in each group

You're editing your response. Sharing this URL allows others to also edit your response.

FILL IN A NEW RESPONSE

22-i) Restate study questions and summarize the answers suggested by the data, starting with primary outcomes and process outcomes (use)

Restate study questions and summarize the answers suggested by the data, starting with primary outcomes and process outcomes (use).

	1	2	3	4	5	
subitem not at all important	○	○	○	○	○	essential

Does your paper address subitem 22-i? *

Copy and paste relevant sections from the manuscript (include quotes in quotation marks "like this" to indicate direct quotes from your manuscript), or elaborate on this item by providing additional information not in the ms, or briefly explain why the item is not applicable/relevant for your study

"The current study was the first to evaluate the cost-effectiveness and cost-utility of an internet-based relapse prevention program for recurrent MDD. The results of the current study suggest that M-CT added to TAU is not cost-effective compared to TAU alone over a 24-month period."

22-ii) Highlight unanswered new questions, suggest future research

Highlight unanswered new questions, suggest future research.

	1	2	3	4	5	
subitem not at all important	○	○	○	○	○	essential

You're editing your response. Sharing this URL allows others to also edit your response.

FILL IN A NEW RESPONSE

Does your paper address subitem 22-ii?

Copy and paste relevant sections from the manuscript (include quotes in quotation marks "like this" to indicate direct quotes from your manuscript), or elaborate on this item by providing additional information not in the ms, or briefly explain why the item is not applicable/relevant for your study

"Based on their systematic review, Erbe, Eichert, Riper, and Ebert [53] concluded that combining the strenth of both face-to-face and internet-based interventions might be a promising direction, although more studies are needed. In addition, besides examining the (cost) effectiveness of specific internet-based interventions, future studies should focus more on the implementation in clinical practice, taking into account specific barriers (e.g., preferences of individuals and professionals) [54,55]. Furthermore, more information is needed under which circumstances face-to-face or other forms of PCT are cost-effective."

"Therefore, it is important that future studies continue to examine highly accessible, scalable, and (potentially) cost-effective interventions to treat depression including interventions that prevent relapse and recurrence. These studies are needed to inform decisions in mental health care. Since treatment effects can manifest differently over time [48], it is important that these cost-effectiveness studies on face-to-face and internet-based interventions include long follow-up periods."

20) Trial limitations, addressing sources of potential bias, imprecision, and, if relevant, multiplicity of analyses

20-i) Typical limitations in ehealth trials

Typical limitations in ehealth trials: Participants in ehealth trials are rarely blinded. Ehealth trials often look at a multiplicity of outcomes, increasing risk for a Type I error. Discuss biases due to non-use of the intervention/usability issues, biases through informed consent procedures, unexpected events.

	1	2	3	4	5	
subitem not at all important	○	○	○	○	○	essential

You're editing your response. Sharing this URL allows others to also edit your response.

FILL IN A NEW RESPONSE

Does your paper address subitem 20-i? *

Copy and paste relevant sections from the manuscript (include quotes in quotation marks "like this" to indicate direct quotes from your manuscript), or elaborate on this item by providing additional information not in the ms, or briefly explain why the item is not applicable/relevant for your study

"Limitations
Some limitations of this study are important to acknowledge. First, cost data were collected with a self-report questionnaire approximately every 3 months during the 24-month follow-up and therefore a substantial amount of participants missed one or more measurement. To deal with missing data, multiple imputations, which is a recommended strategy to handle missing data in cost-effectiveness studies performed alongside randomized controlled trials [56,57], was used in our main analysis. We can assume the data were at least partly missing at random since baseline characteristics predicted whether the data were missing. Nevertheless, the missing completely at random assumption cannot be proved and it is possible data were missing not at random because drop-out could be related to depressive symptom severity. Because of the amount of missing data, we did not want to rely on a single imputation technique and therefore performed several sensitivity analyses that each handled missing data in a different way. The main analysis, the analysis including all participants, and the analysis incorporating participants for whom at least 50% of the data were available (the latter only regarding QALYs) showed similar results. The analysis including participants with at least 50% data (regarding depression-free days) and complete cases showed higher costs and worse outcomes for M-CT compared to TAU. Multiple imputations are preferred over a complete case analysis because of the potential selection bias that might occur due to missing values. The results of the complete cases and cases with at least 50% of the data do suggest a possible selection bias in drop-out, which is also suggested when inspecting a baseline table displaying only the complete cases and cases with 50% of the data. Altogether, we regard our main analysis as primary. Second, the data were obtained in the Netherlands and therefore generalizability into other countries with other treatment settings is questionable. Third, the cost data and data for the cost-utility analysis were based on retrospective self-report questionnaires which may have affected the reliability. The TiC-P has shown to be a reliable and valid questionnaire for collecting cost data [58]. However, the EQ-5D might be subjected to a possible ceiling effect when estimating changes in QALYs for this group of remitted individuals. Moreover, the EQ-5D refers to the current health state and therefore does not capture all relapses/recurrences during the 24 months of the study."

You're editing your response. Sharing this URL allows others to also edit your response.

FILL IN A NEW RESPONSE

21) Generalisability (external validity, applicability) of the trial findings

NPT: External validity of the trial findings according to the intervention, comparators, patients, and care providers or centers involved in the trial

21-i) Generalizability to other populations

Generalizability to other populations: In particular, discuss generalizability to a general Internet population, outside of a RCT setting, and general patient population, including applicability of the study results for other organizations

	1	2	3	4	5	
subitem not at all important	○	○	○	○	○	essential

Does your paper address subitem 21-i?

Copy and paste relevant sections from the manuscript (include quotes in quotation marks "like this" to indicate direct quotes from your manuscript), or elaborate on this item by providing additional information not in the ms, or briefly explain why the item is not applicable/relevant for your study

"Second, the data were obtained in the Netherlands and therefore generalizability into other countries with other treatment settings is questionable."

21-ii) Discuss if there were elements in the RCT that would be different in a routine application setting

Discuss if there were elements in the RCT that would be different in a routine application setting (e.g., prompts/reminders, more human involvement, training sessions or other co-interventions) and what impact the omission of these elements could have on use, adoption, or outcomes if the intervention is applied outside of a RCT setting.

	1	2	3	4	5	
subitem not at all important	○	○	○	○	○	essential

Does your paper address subitem 21-ii?

Copy and paste relevant sections from the manuscript (include quotes in quotation marks "like this" to indicate direct quotes from your manuscript), or elaborate on this item by providing additional

You're editing your response. Sharing this URL allows others to also edit your response.

FILL IN A NEW RESPONSE

OTHER INFORMATION

23) Registration number and name of trial registry

Does your paper address CONSORT subitem 23? *

Copy and paste relevant sections from the manuscript (include quotes in quotation marks "like this" to indicate direct quotes from your manuscript), or elaborate on this item by providing additional information not in the ms, or briefly explain why the item is not applicable/relevant for your study

"Trial registration: trialregister.nl NTR2503"

24) Where the full trial protocol can be accessed, if available

Does your paper address CONSORT subitem 24? *

Cite a Multimedia Appendix, other reference, or copy and paste relevant sections from the manuscript (include quotes in quotation marks "like this" to indicate direct quotes from your manuscript), or elaborate on this item by providing additional information not in the ms, or briefly explain why the item is not applicable/relevant for your study

The study protocol is accessible via:
Bockting CLH, Kok GD, Van der Kamp L, Smit F, Van Valen E, Schoevers R, et al. Disrupting the rhythm of depression using mobile cognitive therapy for recurrent depression: Randomized controlled trial design and protocol. BMC Psychiatry 2011 Jan;11(12):1-9. PMID:21235774

25) Sources of funding and other support (such as supply of drugs), role of funders

You're editing your response. Sharing this URL allows others to also edit your response.

FILL IN A NEW RESPONSE

Does your paper address CONSORT subitem 25? *

Copy and paste relevant sections from the manuscript (include quotes in quotation marks "like this" to indicate direct quotes from your manuscript), or elaborate on this item by providing additional information not in the ms, or briefly explain why the item is not applicable/relevant for your study

"The Netherlands Organisation for Health Research and Development (ZONMW, Department of Disease Management and Chronic Illnesses, grant number 300020014) funded this RCT."

X27) Conflicts of Interest (not a CONSORT item)

X27-i) State the relation of the study team towards the system being evaluated

In addition to the usual declaration of interests (financial or otherwise), also state the relation of the study team towards the system being evaluated, i.e., state if the authors/evaluators are distinct from or identical with the developers/sponsors of the intervention.

	1	2	3	4	5	
subitem not at all important	○	○	○	○	○	essential

Does your paper address subitem X27-i?

Copy and paste relevant sections from the manuscript (include quotes in quotation marks "like this" to indicate direct quotes from your manuscript), or elaborate on this item by providing additional information not in the ms, or briefly explain why the item is not applicable/relevant for your study

"Conflicts of interest
Claudi L.H. Bockting and Evelien van Valen developed M-CT that was integrated in the platform of the Trimbos Institute in collaboration with Filip Smit. No other disclosures are reported."

About the CONSORT EHEALTH checklist

You're editing your response. Sharing this URL allows others to also edit your response.

FILL IN A NEW RESPONSE

As a result of using this checklist, did you make changes in your manuscript? *

- ( ) yes, major changes
- (x) yes, minor changes
- ( ) no

What were the most important changes you made as a result of using this checklist?

Details in the methods regarding the online intervention.

How much time did you spend on going through the checklist INCLUDING making changes in your manuscript *

4 hours

As a result of using this checklist, do you think your manuscript has improved? *

- (x) yes
- ( ) no
- ( ) Other:

Would you like to become involved in the CONSORT EHEALTH group?

This would involve for example becoming involved in participating in a workshop and writing an "Explanation and Elaboration" document

- ( ) yes
- (x) no

You're editing your response. Sharing this URL allows others to also edit your response.

FILL IN A NEW RESPONSE

Any other comments or questions on CONSORT EHEALTH

Your answer

STOP - Save this form as PDF before you click submit

To generate a record that you filled in this form, we recommend to generate a PDF of this page (on a Mac, simply select "print" and then select "print as PDF") before you submit it.

When you submit your (revised) paper to JMIR, please upload the PDF as supplementary file.

Don't worry if some text in the textboxes is cut off, as we still have the complete information in our database. Thank you!

Final step: Click submit !

Click submit so we have your answers in our database!

SUBMIT

Never submit passwords through Google Forms.

This content is neither created nor endorsed by Google. Report Abuse - Terms of Service - Additional Terms

Google Forms

You're editing your response. Sharing this URL allows others to also edit your response.

FILL IN A NEW RESPONSE
