# Supplementary figures and images for: Economic Evaluation of an Internet-Based Preventive Cognitive Therapy With Minimal Therapist Support for Recurrent Depression: Randomized Controlled Trial
Source: J Med Internet Res. 2018 Nov 26;20(11):e10437. doi: 10.2196/10437 (PMC6288594; doi:10.2196/10437)

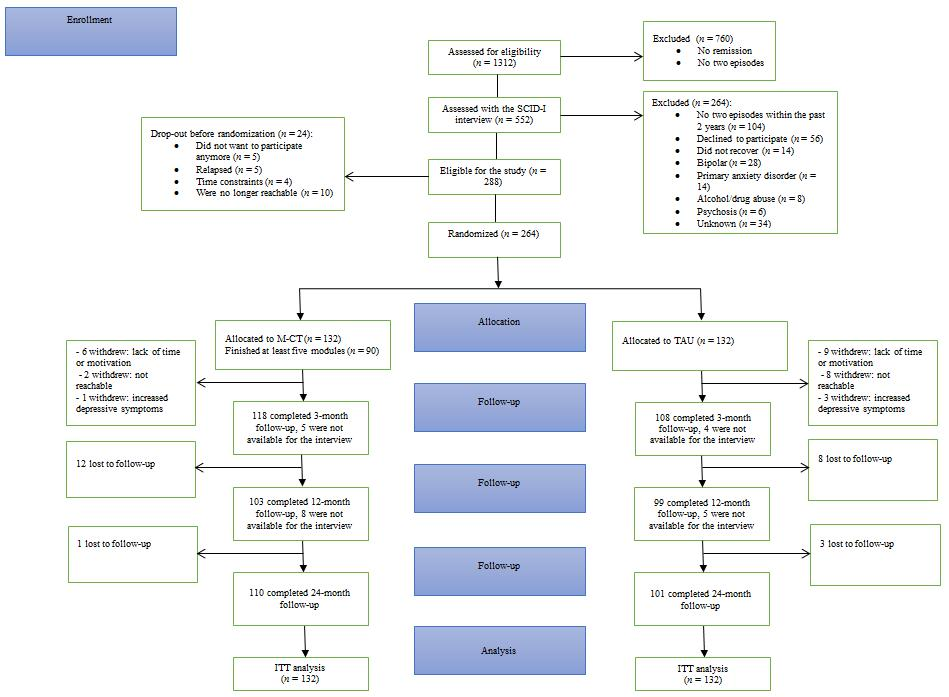

Supplement: Multimedia Appendix 3 [file jmir_v20i11e10437_app3.png]
