# Supplementary material for: Economic Evaluation of an Internet-Based Preventive Cognitive Therapy With Minimal Therapist Support for Recurrent Depression: Randomized Controlled Trial
Source: J Med Internet Res. 2018 Nov 26;20(11):e10437. doi: 10.2196/10437 (PMC6288594; doi:10.2196/10437)
Supplement: Multimedia Appendix 4 [file jmir_v20i11e10437_app4.pdf]

Cumulative costs inside and outside the health care sector over 24 months

Cost types	M-CT (n = 99)		TAU (n = 96)	
	Mean costs (SD)	%[a]	Mean costs (SD)	%[a]
**Health care costs**				
Mobile CT	73 (19)	94	-	0
Psychiatrist	238 (688)	23	93 (262)	19
Psychologist	231 (893)	23	207 (760)	21
Psychotherapist	118 (548)	11	106 (410)	10
Social psychiatric nurse	12 (86)	3	18 (106)	5
Social worker	10 (69)	3	9 (60)	3
Self-help groups	3 (22)	3	18 (114)	6
Various MHC[b] services	590 (1592)	30	879 (1815)	39
Polyclinic care	240 (592)	46	246 (415)	53
CAD[c]	-	0	34 (297)	2
General practitioner	202 (221)	87	156 (162)	79
Company doctor	87 (233)	19	45 (157)	14
Physiotherapist	378 (770)	46	285 (576)	48
Alternative health care	110 (273)	28	209 (450)	35
Antidepressants	52 (144)	46	68 (154)	51
Day care	186 (1719)	3	168 (1168)	4
Hospital admissions	361 (1551)	10	741 (3897)	11
**Patient and family costs**				
Informal care	355 (1070)	43	272 (915)	45
Travel expenses	81 (163)	77	81 (134)	76
(Psychiatric) homecare	29 (166)	3	73 (286)	3

Home care	66 (283)	10	74 (288)	13
**Productivity losses**				
Unpaid work	41 (361)	5	4 (16)	7
Absenteeism (paid work)	1998 (5438)	32	1542 (5744)	28
Presenteeism (paid work)	424 (1442)	38	313 (892)	34

The costs are displayed for participants with at least one cost measurement during follow-up.

[a]Percentage of participants using the cost types concerned

[b]Mental health care services

[c]Consultation office for Alcohol and Drug addiction
